# A systematic molecular circuit design method for gene networks under biochemical time delays and molecular noises

**DOI:** 10.1186/1752-0509-2-103

**Published:** 2008-11-27

**Authors:** Bor-Sen Chen, Yu-Te Chang

**Affiliations:** 1 Lab of Control and Systems Biology, Department of Electrical Engineering, National Tsing Hua University, Hsinchu, Taiwan 30013, ROC

## Abstract

**Background:**

Gene networks in nanoscale are of nonlinear stochastic process. Time delays are common and substantial in these biochemical processes due to gene transcription, translation, posttranslation protein modification and diffusion. Molecular noises in gene networks come from intrinsic fluctuations, transmitted noise from upstream genes, and the global noise affecting all genes. Knowledge of molecular noise filtering and biochemical process delay compensation in gene networks is crucial to understand the signal processing in gene networks and the design of noise-tolerant and delay-robust gene circuits for synthetic biology.

**Results:**

A nonlinear stochastic dynamic model with multiple time delays is proposed for describing a gene network under process delays, intrinsic molecular fluctuations, and extrinsic molecular noises. Then, the stochastic biochemical processing scheme of gene regulatory networks for attenuating these molecular noises and compensating process delays is investigated from the nonlinear signal processing perspective. In order to improve the robust stability for delay toleration and noise filtering, a robust gene circuit for nonlinear stochastic time-delay gene networks is engineered based on the nonlinear robust *H*_∞ _stochastic filtering scheme. Further, in order to avoid solving these complicated noise-tolerant and delay-robust design problems, based on Takagi-Sugeno (T-S) fuzzy time-delay model and linear matrix inequalities (LMIs) technique, a systematic gene circuit design method is proposed to simplify the design procedure.

**Conclusion:**

The proposed gene circuit design method has much potential for application to systems biology, synthetic biology and drug design when a gene regulatory network has to be designed for improving its robust stability and filtering ability of disease-perturbed gene network or when a synthetic gene network needs to perform robustly under process delays and molecular noises.

## Background

Gene expression involves a series of molecular events, which include binding of regulators, transcription, splicing, translation, posttranslation modification and diffusion. As each of these molecular events is subject to significant biochemical process delays, intrinsic fluctuations, and extrinsic disturbances, gene expression is best viewed as a nonlinear stochastic process with multiple time delays [1-4]. Even in cases where population measurements are regular and reproducible, single-cell measurements often display significant heterogeneity [1]. In general, these observations suggest that the molecular events underlying cellular physiology at the nanomolar scale are easily subject to fluctuations and biochemical process delays so that we have to propose a nonlinear stochastic model with multiple time delays for gene expression and biochemistry [2-4]. Therefore, molecular noise processing and delay compensation in nonlinear stochastic gene network with process delays is an important topic to understanding how cells function and process information when the underlying molecular events are random with biochemical process delays. As pointed out in [1,3,4], this topic is one of the most challenging and fascinating problems for systems biologists, since it opens questions in physiology, development and evolutionary biology.

When the underlying molecular events are basically random and time-delayed, how does the physiology of the cell remain highly orchestrated and robust? Most cellular events are orderly and precisely regulated in spite of the stochastic function and process delays of gene regulatory circuits within cells [1,5,6]. Without consideration of biochemical process delay, a stochastic differential equation or the Langevin equation has recently been employed to describe the molecular fluctuation in gene networks [3,7]. Many algorithms have been developed for simulating the Langevin equation to calculate the probability density function [5,6,8,9]. The Fokker-Plank equation is employed to describe the evolution of the probability function [1,10]. Most researchers analyze these stochastic models, using the Monte-Carlo method, such as Gillespie algorithm, StockSim algorithm and so on to describe the evolution of biochemical networks via the discrete stochastic model. Even these modeling tools allow us to address questions concerning intracellular noise. However, if biochemical process delays have not been considered in the dynamic model of biochemical gene networks, an engineered biochemical network based on this model may lead to fluctuation, oscillation or even blowing up [11]. Therefore, biochemical process delays and molecular noises must be considered in the dynamic model to mimic the realistic cellular behaviors of biochemical gene network in cell. Recently, it is also found that most newly synthetic networks are non-functioning and need tuning owing to process delays, parameter fluctuations, due to thermal fluctuation, gene expression noise, mutation and extrinsic noises, due to changing extra cellular environments, interactions with cellular context [12]. Therefore, a systematic design method for noise-tolerant and delay-robust gene networks is an important topic in systems biology and synthetic biology for an engineered gene network to work properly in host cell [13].

In recent years, many researchers have found it useful to invoke analogies from signal processing when investigating molecular noise. In this situation, a biochemical pathway is viewed as an analogy filter and classified in terms of its frequency response from the signal processing perspective. In terms of signal processing, the biochemical pathways function as low-pass filters, as they transduce low-frequency signals and attenuate high-frequency signals. In addition to intrinsic chemical damping, negative feedback [14,15], integral feedback [16], and many other simple mechanisms such as redundancy mechanisms are found to attenuate molecular noise in biochemical systems. It is also found that the effect of molecular noise is also amplified in some sense by autocatalytic mechanisms (positive feedback) to give rise to population heterogeneity (and so diversity). Even some of the elementary mechanisms for molecular noise attenuation and amplification enumerated above seem simple and identifiable. However, elementary mechanisms typically do not function in isolation; rather they interact in complex gene networks involving multiple feedback loops. Although it is straightforward to understand how a single feedback loop shapes molecular noise, it is far more difficult to understand composite behavior of multiple mechanisms interconnected in complex architecture of gene networks [1,8,9]. These molecular noise-related problems are called molecular noise filtering problems of gene network by molecular biologists [1-3,17-19]. The analysis of molecular noise attenuation and amplification of gene regulatory network without process delays has been recently discussed from the stochastic system point of view [17]. At present, though theoretical and computational tools exist for analyzing the filtering properties of a given network, as pointed out by Rao et al. in [1], no good theory exists for identifying all possible systematic mechanisms that generate robust networks to compensate biochemical process delays and to attenuate molecular noises simultaneously.

It is clear that complex gene networks are able to function reliably despite inherent noise attributable to molecular fluctuations and unavoidable time delays due to biochemical process. Robustness has been hypothesized as an intrinsic property of intracellular networks. Although robustness is often studied independent of noise and time delay, the two problems are related. When studying robustness, the typical question is how sensitive the behavior of a gene network is to kinetic parameter perturbations in the model [13]. As these parameter perturbations are subject to molecular fluctuations, a molecular noise-resistant network is likely to be robust. Nevertheless, a gene network that is insensitive to kinetic parameter perturbations may still be sensitive to extrinsic molecular noises. A robust filtering of biochemical networks under parameter perturbations has been discussed based on the global linearization technique in [20]. However, this method is only with S-systems and without consideration of process delays. In [21], based on S-system model [13], a robust circuit design is proposed for nonlinear deterministic biochemical networks from steady state approach and without considering process delay. The robust stabilization method for gene network against molecular noises has been discussed in [22] without consideration of time-delays in biochemical processes.

Time delays are common and substantial in biochemical process. They can protect biochemical systems against transient loss of input signal. Delay for proofreading is widely employed to increase the fidelity of recognition to provide security against misrecognition. Further delay can filter the non-beneficial pulses [11]. However, time-delays may play a negative role in stability of gene networks. In [23,24], the concepts of sensitive edge and robust edge are used to analyze the robustness and fragility of synchronization. Then the authors want to increase the robustness of synchronization by its associated feedback loops to protect against attacks to the complex networks. Further, the robust synchronization designs of complex time-delayed networks are also discussed in [25,26] to increase the robustness of synchronization to tolerate time-delays and protect against attacks to the networks. In this study, the random parameter fluctuations are modeled as state-dependent intrinsic molecular fluctuations of the stochastic gene network model in which multiple-time delays of biochemical process is also considered to mimic the process delays in gene regulation processes. Both the upstream molecular noise and the global molecular noise affecting all genes are modeled as extrinsic molecular noises. The robust molecular noise attenuation problem will be systematically discussed for gene networks with multiple process delays from the nonlinear *H*_∞ _stochastic signal-processing perspective.

The *H*_∞ _filtering theory has been developed for state estimation of nonlinear stochastic signal processing to investigate the noise attenuation problem via minimizing the worst-case effect of noise on the filtering error [27,28] and the *H*_∞ _control theory has been developed to robustly stabilize the nonlinear stochastic system under parameter perturbations [29,30]. However, the time delay of the stochastic process is not considered. Recently, various time-delay control designs for nonlinear systems and fuzzy systems have been discussed in [25,26,31,32]. In this study, the robust nonlinear stochastic *H*_∞ _filtering theory is applied to discuss the robust stability and noise filtering problems of nonlinear stochastic gene networks under both multiple-time delays due to biochemical process delays and stochastic intrinsic molecular noise due to parameter fluctuations. Furthermore, the filtering ability of extrinsic molecular noises will be also investigated at each gene of gene network from the *H*_∞ _filtering point of view. For the biotechnological purpose or drug design purpose, if robust stability is required to tolerate a prescribed range of intrinsic parameter variations and the filtering ability of gene network to filter extrinsic molecular noises from the environment has to be improved, a robust gene circuit design scheme needs to be developed for gene networks from the nonlinear noise filtering perspective to achieve the design purpose via transfection and transformation biotechnologies. For recent metabolic engineering and future synthetic biology [7], if a synthetic gene regulatory network is required to work near a desired equilibrium point in an environment with process delays, intrinsic molecular fluctuations, and extrinsic molecular noises, a robust control circuit design is necessary to improve the robust stability and molecular noise filtering ability of the synthetic gene network to achieve its required performance. This robust filtering design method will be potential for robust gene circuit design in future, from which gene therapy and drug design could be developed. However, the drawback of these nonlinear stochastic filtering approaches needs to solve a nonlinear differential Hamilton-Jacobi inequality (HJI) for the robust stabilization and filtering design of gene networks. In general, we still have no analytic solution or numerical solution for HJI, except for some simple cases. In this study, to avoid solving HJI, a fuzzy interpolation method is employed to interpolate several local linear gene networks at different operation points to approach the nonlinear gene network to simplify the design produce of robust stabilization and filtering of gene networks so that the robust gene circuit design could be easily designed and implemented.

In recent years, Takagi-Sugeno (T-S) fuzzy systems have efficiently interpolated several local linear systems via fuzzy bases to approximate a nonlinear system [33,34]. Therefore, a T-S fuzzy stochastic gene network is employed to approximate a nonlinear stochastic gene network with time delays by interpolating several local linear stochastic gene networks with time delays at different operation points of the nonlinear stochastic gene network. In this study, based on fuzzy interpolation approximation, the robust stabilization problem for tolerating process delays and intrinsic molecular fluctuations and *H*_∞ _filtering design problem for attenuating extrinsic molecular noises of gene networks can be efficiently solved via a set of linear matrix inequalities (LMIs) developed from a set of fuzzy local linear stochastic systems. These LMIs could be easily solved via LMI toolbox in Matlab [35] to simplify the design procedure. Furthermore, based on fuzzy approximation method, the circuit design procedure for robust stability and noise filtering of a nonlinear stochastic gene network with process delay could be simplified from the linear gene network design point of view. In this situation, more biological robust stabilization and filtering insight could be investigated from the linear system point of view. We found that if the eigenvalues of these local linear gene networks are in the far left-hand side of the complex domain (i.e., with more negative real parts or more stable), then the gene network will tolerate more process delays and intrinsic molecular fluctuations, and could also filter more extrinsic molecular noises to achieve the design purpose of gene networks. The robust stabilization and *H*_∞ _molecular noise filtering circuit design could be achieved by shifting the eigenvalues of fuzzy linear gene networks to the far left-hand side of the complex domain through engineering appropriate negative feedback loops via the proposed robust gene circuit design method for nonlinear stochastic time-delayed gene networks.

In general, the proposed robust filter design in nonlinear stochastic gene network is different from the conventional robust fuzzy *H*_∞ _filter design for engineering control or signal processing systems [27-31]. In the conventional fuzzy *H*_∞ _filter design [27-29], a dynamic fuzzy estimator is employed to estimate system state variables from the noisy measurement data, which needs a very complicated computation for state estimation at any time instant. In this study, only some gene circuits are implemented (or embedded) directly in gene network to achieve robust stabilization to tolerate process delay and intrinsic molecular fluctuations and to filter the extrinsic disturbances. Therefore, the proposed robust circuit design is different from the conventional control and filter designs in control engineering and signal processing in [27-31]. Gene circuits are implemented by inserting the transcription factor (TF) binding site of regulatory gene product, i.e. the binding site of protein produced by regulatory gene, to the promoter region of regulated gene by the techniques of transfection and transformation [7,14]. The kinetic parameters of gene circuits of molecular noise filter are proportional to the length of binding sites of TF inserted to the promoter region of regulated genes and will be specified by the designer. In order to simplify the design procedure, only a few gene circuits are implemented. In our design procedure, the T-S fuzzy approximation method is only employed to simplify the specification of kinetic parameters of gene circuit of molecular noise filter, i.e., the specification procedure of lengths of inserted binding sites of TF produced by regulatory genes in gene circuit design procedure. Recent experimental advances in sequencing, genetic engineering and bionanotechnology will make this engineered circuit implementation feasible in near future [7,36-41].

Because disease may perturb the normal gene regulatory network through genetic perturbation and/or by pathological environmental molecular noise such as infection agents or chemical carcinogens [42], in future synthetic biology [7,12,19] and systems biology [13,36,42], we could improve the robust stability and molecular noise filtering ability of gene network for drug design and gene therapy by the proposed gene circuit design method via transfection and transformation biotechnologies, so that a designed gene network could be protected from the influence of process delays, intrinsic molecular fluctuations and environmental molecular noises, and thus work more reliably. Finally, two design examples in silico are given to illustrate the design procedure and to validate the performance of the proposed robust *H*_∞ _gene circuit design method for nonlinear stochastic gene networks under process delays, intrinsic molecular fluctuations and extrinsic molecular noises.

## Results

Firstly, a nonlinear stochastic time-delayed model is developed to mimic the realistic dynamic behavior of a gene network under process delays, random intrinsic and extrinsic molecular noises. Then the robust stability and the filtering ability of nonlinear stochastic time-delayed gene network are discussed from the *H*_∞ _signal processing perspective. Further, in order to improve the robust stability and filtering ability to tolerate more parameter fluctuations, process delays and to attenuate much environmental molecular noises, based on fuzzy approximation and LMI technique, a systematic design method is proposed for nonlinear stochastic time-delayed gene networks. Finally, a simple design procedure is developed for the proposed robust gene circuit design method. These results are described in the following subsections in detail.

### Robust Stability and Filtering Ability Analysis for Nonlinear Stochastic Time-Delayed Gene Networks

For the convenience of illustration, the following linear gene network with process delay is introduced first,

(1) $$\frac{d}{dx}x(t)=A_{0}x(t)+\sum_{k=1}^{m}A_{k}x(t-\tau_{k}),$$

**Figure 1:**
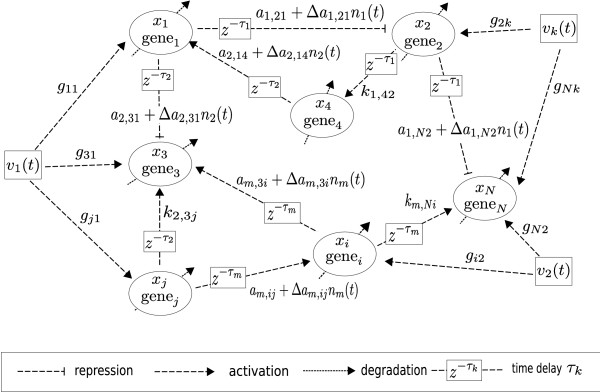
**A linear gene regulatory network of *N *genes under process delays, intrinsic molecular fluctuation Δ*a*_*k*,*ij*_*n*_*k*_(*t*) and extrinsic molecular noise *v*(*t*)**.

*x*(*t*) = *x*_0_(*t*)∀*t *∈ [-*τ*, 0], where *x*(*t*) = [*x*_1_(*t*),...,*x*_*N*_(*t*)]^*T *^denotes the vector of mRNA concentrations of *N *genes; the maximum time delay *τ *= max{*τ*_*k*_, *k *∈ [1, *m*]}; *A*_0 _and *A*_*k *_denote the real-time and delay-time kinetic interaction matrices among these genes, respectively. For example, *x*(*t*) is the mRNA expression vector of *N *genes; the diagonal component *a*_0,*ii *_= -*λ*_*i *_of *A*_0 _denotes the degradation of mRNA of *i*-th gene with decay rate *λ*_*i*_; the *i*, *j *component *a*_*k*,*ij *_of *A*_*k *_denotes the regulatory interaction from gene *j *to gene *i *to activate or repress gene *i *with time delay *τ*_*k*_. The delay time *τ*_*k *_may be due to the process time of gene transcription, translation, posttranslation protein modification and diffusion, which are needed for regulatory genes to product proteins (e.g. transcription factors TFs) and then convey them to their target genes (see Fig. 1). In general, in real biochemical systems, these biochemical process delays may be very long and can not be neglected in biochemical dynamic models, especially for practical gene network design applications.

Suppose the linear gene network in (1) suffers from intrinsic molecular fluctuations so that *a*_*k*,*ij *_→ *a*_*k*,*ij *_+ Δ*a*_*k*,*ij*_*n*_*k*_(*t*), *k *= 0,1,...,*m*, where Δ*a*_*k*,*ij *_denotes the amplitude of the stochastic kinetic fluctuation and *n*_*k*_(*t*) is a white noise with zero mean and unit variance, i.e., Δ*a*_*k*,*ij *_denotes the deterministic part of fluctuations and *n*_*k*_(*t*) absorbs the stochastic property of intrinsic molecular fluctuations (see Fig. 1). Intrinsic molecular fluctuations are mainly attributed to thermal fluctuation and random molecular events in the transcription, splicing, and translation processes of gene expression [1-3]. The covariances of stochastic intrinsic molecular fluctuations Δ*a*_*k*,*ij*_*n*_*k*_(*t*), *k *= 0, 1,...,*m*, are as follows

(2) $$Cov(\Delta a_{k,ij}n_{k}(t_{1}),\Delta a_{k,ij}n_{k}(t_{2}))={\left(\Delta a_{k,ij}\right)}^{2}\delta_{t_{1},t_{2}}$$

for *k *= 0, 1,...,*m*, where $\delta_{t_{1},t_{2}}$ denotes the delta function as follows

$$\delta_{t_{1},t_{2}}=\{\begin{array}{ll} 1 & \text{for }t_{1}=t_{2}\\ 0 & \text{for }t_{1}\neq t_{2} \end{array}$$

According to the above analysis, the gene network under stochastic molecular fluctuations can be represented as

(3) $$\frac{d}{dt}x(t)=(A_{0}+B_{0}n_{0}(t))x(t)+\sum_{k=1}^{m}\left(A_{k}+B_{k}n_{k}(t))x(t-\tau_{k}\right)$$

where the fluctuation matrices *B*_*k *_are given by

$$\begin{array}{cc} B_{k}=\left[\begin{array}{ccc} \Delta a_{k,11} & \cdots & \Delta a_{k,1N}\\ \vdots & \Delta a_{k,ij} & \vdots \\ \Delta a_{k,N1} & \cdots & \Delta a_{k,NN} \end{array}\right], & k=0,1,...,m \end{array}.$$

If *a*_*k*,*ij *_is free of fluctuation, then Δ*a*_*k*,*ij *_= 0 in *B*_*k*_. By the Itô stochastic differential equation, the stochastic gene network equation in (3) is equivalent to the following stochastic process [27,29,31]

(4) $$dx(t)=A_{0}x(t)dt+\sum_{k=1}^{m}A_{k}x(t-\tau_{k})dt+B_{0}x(t)dW_{0}(t)+\sum_{k=1}^{m}B_{k}x(t-\tau_{k})dW_{k}(t)$$

where *W*_*k*_(*t*) is a standard Wiener process or Brownian motion with *dW*_*k*_(*t*) = *n*_*k*_(*t*)*dt*.

Actually, in real gene networks, the dynamic gene regulatory equations are always nonlinear. In this situation, the linear dynamic gene network regulatory equation in (4) should be modified as the following Langevin nonlinear stochastic equation with multiple time delays [1,3,10]

(5) $$dx(t)=f_{0}(x(t))dt+\sum_{k=1}^{m}f_{k}(x(t-\tau_{k}))dt+h_{0}(x(t))dW_{0}(t)+\sum_{k=1}^{m}h_{k}(x(t-\tau_{k}))dW_{k}(t)$$

where the first two terms on the right-hand side denote the nominal nonlinear interactions of gene network and the last two terms denote the effect of intrinsic molecular fluctuations on the gene network, which are state-dependent and will influence the stability of nominal gene network.

**Remark 1 ***If the Langevin equation in (5) is linearized at an operation point, it should be a linear stochastic gene network as (4)*.

For simplicity, the linear stochastic gene network in (4) and nonlinear stochastic gene network in (5) will be reformulated, respectively, as follows

(6) $$dx(t)=\sum_{k=0}^{m}A_{k}x(t-\tau_{k})dt+\sum_{k=0}^{m}B_{k}x(t-\tau_{k})dW_{k}(t)$$

and

(7) $$dx(t)=\sum_{k=0}^{m}f_{k}(x(t-\tau_{k}))dt+\sum_{k=0}^{m}h_{k}(x(t-\tau_{k}))dW_{k}(t)$$

where *τ*_0 _= 0 and *τ*_*k *_> 0, *k *= 1,...,*m*, denote the corresponding time delays.

We say that the stochastic gene network is asymptotically stable in probability at the equilibrium point *x *= 0 if there exists a Lyapunov (power-like) function *V*(*x*(*t*)) > 0, with *V*(0) = 0, in the neighborhood of the equilibrium point such that the expectation of the derivative of *V*(*x*(*t*)), $E\frac{d}{dt}V(x(t))<0$, i.e., the power of the gene network is decreasing. In general, there exist many equilibrium points for nonlinear gene networks in (7). Gene networks perform their biochemical function within some local region of an equilibrium point, which is called the phenotype of the gene network. For the convenience of design, only the robust stability of the equilibrium point at *x *= 0 is discussed for the nonlinear stochastic gene network in (7). If the interested equilibrium point (steady state) of the nonlinear time-delayed gene network is not at *x *= 0, the origin should be shifted to the equilibrium point or the steady state. In this situation, the robust stabilization and filtering ability at the origin is equivalent to the stabilization and filtering ability at the interested equilibrium point of the gene network. According to the stochastic gene network model in (7) and the nonlinear stochastic stability theory in [27,30,31], the robust stability and molecular noise filtering ability of stochastic time-delayed gene networks are analyzed in the following propositions.

**Proposition 1 ***Assume there exists a positive Lyapunov function V*(*x*(*t*)) > 0 and *V *(0) = 0 *satisfying the following second-order Hamilton-Jacobi inequality (HJI)*

(8) $${\left(\frac{\partial V(x(t))}{\partial x}\right)}^{T}\sum_{k=0}^{m}f_{k}(x(t-\tau_{k}))+\sum_{k=0}^{m}\frac{1}{2}h_{k}^{T}(x(t-\tau_{k}))\frac{\partial^{2}V(x(t))}{\partial x^{2}}h_{k}(x(t-\tau_{k}))<0$$

*for all nonzero x*(*t*) ∈ *R*^*N*^, *then the equilibrium point x = 0 of the nonlinear stochastic gene network in (7) is asymptotically stable in probability, i.e., the effects of the time delays and intrinsic molecular fluctuations $\sum_{k=0}^{m}h_{k}(x(t-\tau_{k}))dW_{k}(t)$ could be tolerated by the gene network*.

**Proof**. See Appendix A.   ■

**Remark 2 ***The last diffusion term in (8) is from the intrinsic molecular noises in (7). If the gene network is free of intrinsic molecular fluctuations, then the asymptotic stability of the equilibrium point is only to check the existence of V*(*x*(*t*)) > 0 *in the following inequality without diffusion terms*

(9) $${\left(\frac{\partial V(x(t))}{\partial x}\right)}^{T}\sum_{k=0}^{m}f_{k}(x(t-\tau_{k}))<0$$

**Proposition 2 ***For the linear stochastic gene network with process delays in (6), we could choose the Lyapunov function as *[35]

$$V(x(t))=x^{T}(t)Px(t)+\sum_{k=1}^{m}\int_{t-\tau_{k}}^{t}x^{T}(s)Q_{k}x(s)ds$$

*for P = P*^*T *^> 0 *and *$Q_{k}=Q_{k}^{T}>0$ > 0, *i *= 1,...,*m. Then the robust stability problem in Proposition 1 is reduced to check whether or not the existence of symmetric matrices P *> 0 *and Q*_*k *_> 0, *k *= 1,...,*m, in the following LMI*

(10) $$\left[\begin{array}{cccc} \Xi & PA_{1} & \cdots & PA_{m}\\ A_{1}^{T}P & B_{1}^{T}PB_{1}-Q_{1} & 0 & 0\\ \vdots & 0 & \ddots & 0\\ A_{m}^{T}P & 0 & 0 & B_{m}^{T}PB_{m}-Q_{m} \end{array}\right]<0$$

where $\Xi =A_{0}^{T}P+PA_{0}+B_{0}^{T}PB_{0}+\sum_{k=1}^{m}Q_{k}$.

**Proof**. See Appendix B.   ■

If the linear gene network in (6) is free of intrinsic molecular fluctuations, then the asymptotic stability is only to check whether or not *P *> 0 and *Q*_*k *_> 0, *k *= 1,..., *m*, exist in the following LMI

(11) $$\left[\begin{array}{cccc} A_{0}^{T}P+PA_{0}+\sum_{k=1}^{m}Q_{k} & PA_{1} & \cdots & PA_{m}\\ A_{1}^{T}P & -Q_{1} & 0 & 0\\ \vdots & 0 & \ddots & 0\\ A_{m}^{T}P & 0 & 0 & -Q_{m} \end{array}\right]<0$$

Obviously, the condition for the existence of *P *> 0 and *Q*_*k *_> 0, *k *= 1,...,*m*, in inequality (10) is stricter than that in inequality (11) because *A*_0 _should be more negative than that in inequality (11), i.e., the eigenvalues of *A*_0 _should be in the farther left-hand side of the complex domain (the real parts of all eigenvalues of *A*_0 _should be more negative) or more robustly stable in order to tolerate time delays and intrinsic molecular fluctuations.

In general, a gene network in vivo also suffers from extrinsic molecular noises such as the transmitted noise from upstream genes and the global noise affecting all genes. Therefore, the nonlinear stochastic gene regulatory equation with process delays in (7) should be modified in the following to mimic the realistic dynamic behavior in its host cell.

(12) $$dx(t)=\sum_{k=0}^{m}f_{k}(x(t-\tau_{k}))dt+Gv(t)dt+\sum_{k=0}^{m}h_{k}(x(t-\tau_{k}))dW_{k}(t)$$

where signal vector $v(t)={\left[v_{1}(t),...,v_{n_{v}}(t)\right]}^{T}$ denotes the extrinsic molecular noises and *G *denotes the coupling matrix between the extrinsic molecular noises and the gene network. In general, the intrinsic molecular fluctuations are state-dependent as shown in (6)–(7). Therefore, they will influence the stability of gene network and provide an important topic for discussing the robust stability of gene network at an equilibrium point of interest, i.e. to discuss how much the gene network could tolerate the intrinsic molecular fluctuation $\sum_{k=0}^{m}h_{k}(x(t-\tau_{k}))dW_{k}(t)$ without blowing away from the equilibrium point. Another important topic is how to quantify the ability to filter extrinsic molecular noises *v*(*t*) in the gene network, i.e. the attenuation analysis of *v*(*t*) on *x*(*t*) by the gene network. Such information is crucial for understanding the signal-processing scheme in gene networks, from which the design of molecular noise-tolerant and time delay-compensation gene circuits can be developed to achieve a robust *H*_∞ _noise filtering design for biotechnological purposes.

Therefore, we want to investigate the influence of extrinsic molecular noises *v*(*t*) on the gene *i*, i.e. *x*_*i*_(*t*), which is crucial to the design of noise-tolerant gene circuit in synthetic biology [7]. Let us denote the choice of a gene of interest as

(13) *z*_*o*_(*t*) = *Cx*(*t*)

where the row vector *C *= [0, 0,...0, 1, 0, 0, 0, 0], i.e., all elements of *C *are zero except the *i*-th element being 1, if the *i*-th gene *x*_*i*_(*t*) is the gene of interest in molecular noise filtering. Let us denote the effect of extrinsic molecular noises *v*(*t*) on a gene of interest as follows

$$\frac{{\left(E\int_{0}^{\infty }z_{o}^{T}(t)z_{o}(t)dt\right)}^{1/2}}{{\left(E\int_{0}^{\infty }v^{T}(t)v(t)dt\right)}^{1/2}}\leq \gamma ,$$

or

(14) $$E\int_{0}^{\infty }z_{o}^{T}(t)z_{o}(t)dt\leq \gamma^{2}E\int_{0}^{\infty }v^{T}(t)v(t)dt$$

for all bounded energy noises *v*(*t*). This is the so-called *H*_∞ _noise filtering problem [27], which addresses the effect of extrinsic molecular noises on the gene of interest from the system gain (*L*_2_-gain) point of view. If the extrinsic molecular noises *v*(*t*) are deterministic signals, then the expectation *E *on *v*(*t*) in (14) could be neglected.

Remark 3

*(i) The physical meaning of (14) is that the effect of extrinsic molecular noises v*(*t*) *on z*_*o*_(*t*) *should be less than or equal to γ. If γ < 1, then extrinsic molecular noises v*(*t*) *are filtered at gene i by the gene network. If γ > 1, then it means that extrinsic molecular noises v(t) are amplified at gene i, i.e., gene i is much influenced by external molecular noises v*(*t*) *and may be a weak site of the gene network*.

*(ii) In the conventional control system design and signal processing *[27-32], *the H_∞ _noise filtering criterion in (14) has been employed to design a filter to robustly estimate the state variables from the noisy measurement output, i.e., to specify the filter design parameters to efficiently attenuate the effect of uncertain external disturbance on the state estimation error of the filter *[27,28]. *In this study, we only discuss the effect of external disturbance on the genes of nonlinear time-delayed gene network and then engineer some gene circuits to improve the noise filtering ability of the nonlinear time-delayed gene network to attenuate the effect of external disturbance. Therefore, there are some differences between the proposed noise filtering circuit design in gene network and the conventional H_∞ _filter design to robustly estimate state variables in a signal processing system under external disturbance*.

*(iii) If we want to discuss the effect of extrinsic molecular noises v*(*t*)* on the whole gene network, then C in (13) should be an identity matrix, i.e. C = I and z*_*o*_(*t*) = *x*(*t*).

*(iv) If the initial condition x*(0) ≠ 0, *then the filtering ability in (14) should be modified as follows *[27,28]

(15) $$E\int_{0}^{\infty }z_{0}^{T}(t)z_{o}(t)dt\leq EV(x(0))+\gamma^{2}E\int_{0}^{\infty }v^{T}(t)v(t)dt$$

*for some Lyapunov function V *(*x*(*0*)), *i.e., the energy due to initial condition x(0) should be considered in the H_∞ _filtering performance*.

Then we get the following molecular noise filtering result for the nonlinear stochastic gene network with process delays, intrinsic molecular fluctuations, and extrinsic molecular noises in (12).

**Proposition 3 ***If there exists a positive function V *(*x*(*t*)) > 0 *with V *(0) = 0 *solving the following HJI*

(16) $$\begin{array}{r} {\left(\frac{\partial V(x(t))}{\partial x}\right)}^{T}\sum_{k=0}^{m}f_{k}(x(t-\tau_{k}))+x^{T}(t)C^{T}Cx(t)+\frac{1}{4\gamma^{2}}{\left(\frac{\partial V(x(t))}{\partial x}\right)}^{T}GG^{T}\frac{\partial V(x(t))}{\partial x}\\ +\sum_{k=0}^{m}\frac{1}{2}h_{k}^{T}(x(t-\tau_{k}))\frac{\partial^{2}V(x(t))}{\partial x^{2}}h_{k}(x(t-\tau_{k}))<0 \end{array}$$

*then the influence of molecular noises v*(*t*)* on the gene network is less than γ or equivalently, the molecular noise filtering ability γ in (14) or (15) is achieved in the nonlinear stochastic gene network under process delays, extrinsic molecular noises, and intrinsic molecular fluctuations*.

**Proof**. See Appendix C.   ■

Therefore, the optimal extrinsic molecular noise filtering ability of gene network at the gene of interest is obtained by solving the following constrained optimization

(17) $$\begin{array}{c} \gamma_{0}=\min\gamma \\ \text{subject to }(16) \end{array}$$

i.e., the molecular noise filtering ability *γ*_0 _of *v*(*t*) on gene *i *could be evaluated by the optimal *H*_∞ _filtering ability of gene network via solving the constrained optimization in (17).

Remark 4

*(i) From Propositions 1 and 3, we gain more insight into robust stability and molecular noise filtering of nonlinear stochastic time-delayed gene network from the nonlinear stochastic H_∞ _filtering perspective. The solution for HJI in (16) is stricter than that for HJI in (8), because of two extra terms for the robust filtering of extrinsic molecular noises in (16). i.e., Proposition 3 not only guarantees robust stability against process delay and intrinsic molecular fluctuations, but also achieves a prescribed noise filtering ability γ against extrinsic molecular noises*.

*(ii) In general, a nonlinear stochastic gene network has many equilibrium points (i.e. phenotypes). We are only concerned about the robust stability of one equilibrium point of interest, i.e. the equilibrium point at the steady state of gene networks. Propositions 1 and 3 are true only at the equilibrium point (one phenotype) of the origin, i.e. x*_*e *_= 0. *If we are interested in an equilibrium point x*_*e *_≠ 0 *(i.e. another phenotype), for the convenience of investigation, the origin should be shifted to x*_*e*_, *i.e., the stochastic system in (12) should be modified as*

(18) $$dx^{\prime }(t)=\sum_{k=0}^{m}f_{k}(x^{\prime }(t-\tau_{k})+x_{e})dt+Gv(t)+\sum_{k=0}^{m}h_{k}(x^{\prime }(t-\tau_{k})+x_{e})dW_{k}(t)$$

*where x*'(*t*) = *x*(*t*) - *x*_*e*_. *Therefore, when we are interested in an equilibrium point x*_*e *_≠ 0 *of gene network, the variable x*(*t*) *in HJIs (8) and (16) should be replaced by x'*(*t*) + *x*_*e *_*so that the origin of the coordinate should be shifted to x*_*e*_. *This shift is a one-to-one correspondence *[43]. *Therefore, instead of studying the behavior of gene network in the neighborhood of x*_*e*_, *one can equivalently study the behavior in the neighborhood of the origin x*'(*t*) = 0 *in (18)*.

In general, it is still very difficult to solve the second-order HJI in (8) or (16) with *V *(*x*(*t*)) > 0 and *V *(0) = 0 to guarantee the robust stability of a nonlinear stochastic gene network under process delay and intrinsic molecular fluctuations or to solve the constrained minimization in (17) to get the noise filtering ability *γ*_0 _on extrinsic molecular noises at any gene of the gene network, especially for complex gene networks. In this situation, the T-S fuzzy model is employed to interpolate several linear gene regulatory networks at different operation points to efficiently and globally approximate the stochastic gene networks in (7) and (12). According to the T-S fuzzy model, both the robust stability of a gene network to tolerate process delays and intrinsic molecular fluctuations and the filtering ability of attenuating extrinsic molecular noises of the gene network could be efficiently investigated and calculated via fuzzy interpolation of several local linear gene networks. Finally, if the gene regulatory network cannot tolerate process delays and intrinsic parameter variations and achieve a prescribed noise filtering level *γ *<*γ*_0 _for some biotechnological or drug design purpose, a robust gene circuit control design is developed from the fuzzy robust stabilization and *H*_∞ _filtering theory to achieve the robust stability and to improve the filtering ability of the gene network. The proposed robust *H*_∞ _filtering design method for gene networks is also useful for synthetic biology in the near future, if a synthetic gene network wants to perform reliably under process delays, intrinsic molecular fluctuations, and extrinsic molecular noises.

### Robust *H*_∞ _Gene Circuit Design of Nonlinear Gene Network Under Process Delays and Molecular Noises via Fuzzy Methodology

If the linear stochastic gene network in (6) is not robust enough and cannot tolerate the molecular fluctuations $\sum_{k=0}^{m}B_{k}x(t-\tau_{k})dW_{k}(t)$, which are state-dependent and will affect the stability of gene network. i.e., the perturbative gene network in equation (6) becomes unstable, then a gene circuit design is necessary for the gene network. We want to engineer some feedback gene circuits $\sum_{k=0}^{m}K_{k}x(t-\tau_{k})$ to robustly stabilize the perturbative gene network as follows

(19) $$\begin{array}{c} dx(t)=\sum_{k=0}^{m}(A_{k}x(t-\tau_{k})+K_{k}x(t-\tau_{k}))dt+\sum_{k=0}^{m}B_{k}x(t-\tau_{k})dW_{k}(t) \end{array}$$

(20) $$=\sum_{k=0}^{m}{\bar{A}}_{k}x(t-\tau_{k})dt+\sum_{k=0}^{m}B_{k}x(t-\tau_{k})dW_{k}(t)$$

where ${\bar{A}}_{k}≜A_{k}+K_{k};K_{k},k=0,1,...,m$, are the circuit kinetic parameter matrices of gene circuits to be designed. The element *k*_0,*ij *_of *K*_0 _denotes the circuit parameter to be specified for the engineered gene circuit between gene *j *and gene *i *via transfection and transformation biotechnologies (see Fig. 1). The gene circuit from gene *j *to gene *i *can be implemented by inserting the binding site of gene product *j *(i.e. the protein of gene *j*) into the promoter region of gene *i *so that the protein of gene *j *could bind this inserted binding site to act as a transcription factor (TF) to regulate the gene expression of gene *i*. By inserting strong (long) or weak (short) binding site, we can get a large or small circuit kinetic parameter *k*_0,*ij*_. The inserting of a TF binding site into promoter region or the deleting of a TF binding site from the promoter region can be easily done by using a highly efficient phage-based homologous recombination system, called recombineering [37,38]. Furthermore, the change of decay rates in the diagonal terms of *A*_0 _by the diagonal terms of *K*_0 _can be implemented by the mechanisms and controls of mRNA degradation through elongating or shortening the 3' polyadenylate tail of mRNA [39,40,44-46]. These powerful biotechnologies have been employed to engineer large segments of genomic DNA to generate transgenic and knockout constructs. However, for simplicity and feasibility, only a few feasible gene circuits in *K*_0_*x*(*t*) are considered for gene circuit design in gene network, i.e., the circuit kinetic parameter matrix *K*_0 _has with only a few elements *k*_0,*ij*_. The design principle of *K*_*k*_, *k *= 1,...,*m*, is similar. A more detailed design procedure will be given in the design example in the sequel. Therefore, in the gene circuit design procedure, we engineer some feasible gene circuits *k*_*k*,*ij*_*x*_*j*_(*t *- *τ*_*k*_) for a gene network with adequate *k*_*k*,*ij *_to achieve robust stabilization and filtering design. In the situation, the specification of *k*_*k*,*ij *_for the embedded gene circuits is different from the convenient control or filter design method, which is used to calculate control input or state estimation from state variables or output signals. However, the closed-form solutions for *K*_*k*_, *k *= 0, 1,...,*m*, are diffcult to be obtained. Therefore, some searching algorithms for components of *K*_*k*_, *k *= 0, 1,...,*m*, are needed.

Following the robust stability inequality in (10), we could get the following result.

**Proposition 4 ***The designed gene network in (20) is robustly stable if we could specify K*_*k*_, *k *= 0, 1,...,*m, for the designed gene circuits such that there exist the positive definite symmetric matrices P > 0 and Q*_*k *_> *0, k *= 1,...,*m, solving the following inequality*

(21) $$\left[\begin{array}{cccc} \Xi & P{\bar{A}}_{1} & \cdots & P{\bar{A}}_{m}\\ {\bar{A}}_{1}^{T}P & B_{1}^{T}PB_{1}-Q_{1} & 0 & 0\\ \vdots & 0 & \ddots & 0\\ {\bar{A}}_{m}^{T}P & 0 & 0 & B_{m}^{T}PB_{m}-Q_{m} \end{array}\right]<0$$

*where *$\Xi ={\bar{A}}_{0}^{T}P+P{\bar{A}}_{0}+B_{0}^{T}PB_{0}+\sum_{k=1}^{m}Q_{k}$*and *${\bar{A}}_{k}$ = *A*_*k *_+ *K*_*k*_, i.e., *the robust gene circuit design becomes how to specify K*_*k*_, *k *= 0, 1,...,*m*, *such that there exist the positive symmetric matrices P and Q*_*k*_, *k *= 1,...,*m*, *in LMI (21)*.

**Proof**. The proof is trivial.   ■

**Remark 5 ***In order to make sure the positive concentrations of gene regulation network in (19) or (20), the choice of designed kinetic parameter K*_*k *_*to solve LMI in (21) should guarantee the positive concentrations of gene networks. In general, according to the positive orthant stabilization theory *[35], *the guaranty for the positive concentration of x*(*t*) *is that the off-diagonal entries of *${\bar{A}}_{k}$ = *A*_*k *_+ *K*_*k *_*are all nonnegative. Therefore, the specification of K*_*k *_*to solve LMI should be subjected to this constraint. If these concentration constraint can not be achieved, changes of gene circuits through other loop should be tried*.

If the nonlinear stochastic time-delayed gene network in equation (7) cannot tolerate molecular fluctuations $\sum_{k=0}^{m}h_{k}(x(t-\tau_{k}))dW_{k}(t)$, then we want to design the gene circuits $\sum_{k=0}^{m}K_{k}g_{k}(x(t-\tau_{k}))$ in the following to robustly stabilize the perturbative gene network

(22) $$dx(t)=\sum_{k=1}^{m}\left(f_{k}(x(t-\tau_{k})\right)+K_{k}g_{k}(x(t-\tau_{k})))dt+\sum_{k=1}^{m}h_{k}(x(t-\tau_{k}))dW_{k}(t)$$

where *g*_*k*_(*x*(*t *- *τ*_*k*_)), *k *= 0, 1,...,*m*, denote the nonlinear feedback circuits and the matrices *K*_*k*_, *k *= 0, 1,...,*m*, denote their corresponding circuit kinetic parameters to be specified to robustly stabilize the perturbative gene network. In general, for the nonlinear stochastic gene network with time delays in (22), it is not easy to obtain a systematic design method for *K*_*k*_, *k *= 0,1,...,*m*, to achieve the robust gene circuit design.

The fuzzy dynamic model with time delay has been widely employed to interpolate several local linear time-delayed dynamic models to efficiently approximate a nonlinear time-delayed dynamic system. The T-S fuzzy dynamic model is described by fuzzy If-Then rules and employed here to deal efficiently with the robust *H*_∞ _filtering problem for gene networks under process delay, intrinsic molecular fluctuations and extrinsic molecular noises in (22). The *i*-th rule of the fuzzy model for nonlinear stochastic time-delayed gene network in (22) is proposed as the following form [33,34]:

(23) $$\begin{array}{c} \text{Rule }i:\text{If }z_{1}(t)\text{ is }F_{i1}\text{ and }z_{2}(t)\text{ is }F_{i2}\ldots \text{ and }z_{g}(t)\text{ is }F_{ig},\\ \text{then }dx(t)=\sum_{k=0}^{m}(A_{k,i}x(t-\tau_{k})+K_{k}G_{k,i}x(t-\tau_{k}))dt+\sum_{k=0}^{m}B_{k,i}x(t-\tau_{k})dW_{k}(t) \end{array}$$

for *i *= 1, 2,...,*L*. *F*_*ij *_is the fuzzy set; *A*_*k*,*i*_, *B*_*k*,*i*_, and *G*_*k*,*i *_(*k *= 0, 1,...,*m*) are known constant matrices; *L *is the number of If-Then rules; *z*(*t*) = [*z*_1_(*t*),...,*z*_*g*_(*t*)]^*T *^are the premise variables; *g *is the number of premise variables. If all state variables *x*(*t*) are used as premise variables, then *z*(*t*) = *x*(*t*) and *g *= *N*. The physical meaning of the fuzzy rule i is that if premise variables *z*_1_(*t*), *z*_2_(*t*),...,*z*_*g*_(*t*) are with the fuzzy sets *F*_*i*1_, *F*_*i*2_,...,*F*_*ig*_, then the nonlinear stochastic gene network in (22) can be represented by interpolating the linearized systems in (23). The fuzzy stochastic system in (23) is inferred as follows [33,34]

(24) $$dx(t)=\sum_{i=1}^{L}\mu_{i}(z)\left[\sum_{k=0}^{m}\left(A_{k,i}x(t-\tau_{k}\right)+K_{k}G_{k,i}x(t-\tau_{k}))dt+\sum_{k=0}^{m}B_{k,i}x(t-\tau_{k})dW_{k}(t)\right]$$

where

$$\mu_{i}(z)≜\frac{\prod_{j=1}^{g}F_{ij}(z_{j}(t))}{\sum_{l=1}^{L}\prod_{j=1}^{g}F_{lj}(z_{j}(t))}$$

*F*_*ij*_(*z*_*j*_(*t*)) is the grade of membership of *z*_*j*_(*t*) in *F*_*ij *_or the possibility function of *z*_*j*_(*t*) in *F*_*ij *_and *μ*_*i*_(*z*), for *i *= 1, 2,...,*L*, are called the fuzzy bases. The denominator $\sum_{l=1}^{L}\prod_{j=1}^{g}F_{lj}(z_{j}(t))$ is only for normalization so that the total sum of fuzzy bases $\sum_{i=1}^{L}\mu_{i}(z)=1$. The physical meaning of (24) is that the fuzzy stochastic system interpolates *L *local linear stochastic systems through nonlinear bases *μ*_*i*_(*z*) to approximate the nonlinear stochastic gene network in (22). In this situation, the Langevin stochastic equation in (22) can be represented by the fuzzy interpolatory gene network as follows

(25) $$\begin{array}{c} dx(t)=\sum_{k=0}^{m}(f_{k}(x(t-\tau_{k}))+K_{k}g_{k}(x(t-\tau_{k})))dt+\sum_{k=0}^{m}h_{k}(x(t-\tau_{k}))dW_{k}(t)\\ =\sum_{i=1}^{L}\mu_{i}(z)\left[\sum_{k=0}^{m}(A_{k,i}x(t-\tau_{k})+K_{k}G_{k,i}x(t-\tau_{k}))dt+\sum_{k=0}^{m}B_{k,i}x(t-\tau_{k})dW_{k}(t)\right]\\ +\sum_{k=0}^{m}\Delta f_{k}(x)dt+\sum_{k=0}^{m}K_{k}\Delta g_{k}(x)dt+\sum_{k=0}^{m}\Delta h_{k}(x)dW_{k}(t)\\ =\sum_{i=1}^{L}\mu_{i}(z)\left[\sum_{k=0}^{m}{\bar{A}}_{k,i}x(t-\tau_{k})dt+\sum_{k=0}^{m}B_{k,i}x(t-\tau_{k})dW_{k}(t)\right]\\ +\sum_{k=0}^{m}\Delta f_{k}(x)dt+\sum_{k=0}^{m}K_{k}\Delta g_{k}(x)dt+\sum_{k=0}^{m}\Delta h_{k}(x)dW_{k}(t) \end{array}$$

where ${\bar{A}}_{k,i}≜A_{k,i}+K_{k}G_{k,i}$; Δ*f*_*k*_(*x*), Δ*g*_*k*_(*x*), and Δ*h*_*k*_(*x*) denote fuzzy approximation errors as follows

$$\begin{array}{ll} \Delta f_{k}(x) & ≜\Delta f_{k}(x(t-\tau_{k}))=f_{k}(x(t-\tau_{k}))-\sum_{i=1}^{L}\mu_{i}(z)A_{k,i}x(t-\tau_{k})\\ \Delta g_{k}(x) & ≜\Delta g_{k}(x(t-\tau_{k}))=g_{k}(x(t-\tau_{k}))-\sum_{i=1}^{L}\mu_{i}(z)G_{k,i}x(t-\tau_{k})\\ \Delta h_{k}(x) & ≜\Delta h_{k}(x(t-\tau_{k}))=h_{k}(x(t-\tau_{k}))-\sum_{i=1}^{L}\mu_{i}(z)B_{k,i}x(t-\tau_{k}) \end{array}$$

There are many methods for finding *A*_*k*,*i *_and *B*_*k*,*i*_, *k *= 0, 1,...,*m*, *i *= 1, 2,...,*L*, for fuzzy model identification [33]. We could use fuzzy toolbox in Matlab to find *A*_*k*,*i*_, *B*_*k*,*i*_, and *G*_*k*,*i *_easily. After finding *A*_*k*,*i*_, *B*_*k*,*i*_, and *G*_*k*,*i*_, *k *= 0, 1,...,*m*, *i *= 1, 2,...,*L*, we could easily find the bounds of fuzzy approximation errors Δ*f*_*k*_(*x*), Δ*h*_*k*_(*x*) and Δ*g*_*k*_(*x*) as follows

(26) $$\begin{array}{c} ||\Delta f_{k}(x)|{|}_{2}\leq a_{k}||x(t-\tau_{k})|{|}_{2}\\ ||\Delta h_{k}(x)|{|}_{2}\leq b_{k}||x(t-\tau_{k})|{|}_{2}\\ ||\Delta g_{k}(x)|{|}_{2}\leq c_{k}||x(t-\tau_{k})|{|}_{2} \end{array}$$

for some positive constants *a*_*k*_, *b*_*k*_, *c*_*k*_, *k *= 0, 1,...,*m*, where $||x(t-\tau_{k})|{|}_{2}≜{\left(x_{1}^{2}(t-\tau_{k})+x_{2}^{2}(t-\tau_{k})+\cdots +x_{N}^{2}(t-t_{k})\right)}^{1/2}$, e.g. if *k *= 0 then $||x(t-\tau_{0})|{|}_{2}=||x(t)|{|}_{2}={\left(x_{1}^{2}(t)+x_{2}^{2}(t)+\cdots +x_{N}^{2}(t)\right)}^{1/2}$ with *τ*_0 _= 0 in (7).

According to the above fuzzy approximation method, we get the following result.

**Proposition 5 ***The nonlinear stochastic gene network with time delays in (22) is robustly stabilizable by gene circuits K*_*k*_*g*_*k*_(*x*(*t *- *τ*_*k*_)), *k *= 0, 1,...,*m*, *if there exist the positive definite symmetric matrices P *> 0 and *Q*_*k *_> 0, *k *= 1,...,*m*, solving the following LMIs

(27) $$\left[\begin{array}{cccc} \Xi_{11,i} & \Xi_{12,i} & \Xi_{13,i} & \Xi_{14,i}\\ \Xi_{21,i} & -\Xi_{22,i} & 0 & 0\\ \Xi_{31,i} & 0 & -\Xi_{33,i} & 0\\ \Xi_{41,i} & 0 & 0 & -\Xi_{44,i} \end{array}\right]<0$$

for all *i *= 1,⋯,*L*, and *P *<*βI*, where

$$\begin{array}{l} \Xi_{11,i}=\left[\begin{array}{cc} \Theta_{11,i} & \Theta_{12,i}\\ \Theta_{21,i} & \Theta_{22,i} \end{array}\right],\Xi_{12,i}=\left[\begin{array}{ccc} P & \cdots & P \end{array}\right],\Xi_{21,i}=\Xi_{12,i}^{T}\\ \Xi_{22,i}=\left[\begin{array}{ccc} \alpha_{0,1}I & 0 & 0\\ 0 & \ddots & 0\\ 0 & 0 & \alpha_{m,1}I \end{array}\right],\Xi_{13,i}=\left[\begin{array}{ccc} B_{0,i}^{T}P & 0 & 0\\ 0 & \ddots & 0\\ 0 & 0 & B_{m,i}^{T}P \end{array}\right]\\ \Xi_{33,i}=\left[\begin{array}{ccc} \alpha_{0,2}I & 0 & 0\\ 0 & \ddots & 0\\ 0 & 0 & \alpha_{m,2}I \end{array}\right],\Xi_{14,i}=\left[\begin{array}{ccc} PK_{0} & \cdots & PK_{m} \end{array}\right]\\ \Xi_{44,i}=\left[\begin{array}{ccc} \alpha_{0,3}I & 0 & 0\\ 0 & \ddots & 0\\ 0 & 0 & \alpha_{m,3}I \end{array}\right],\Xi_{31,i}=\Xi_{13,i}^{T},\Xi_{41,i}=\Xi_{14,i}^{T}\\ \Theta_{11,i}=P{\bar{A}}_{0,i}+{\bar{A}}_{0,i}^{T}P+{\bar{\alpha }}_{0}I+B_{0,i}^{T}PB_{0,i}+\sum_{k=1}^{m}Q_{k}\\ \Theta_{12,i}=\left[\begin{array}{ccc} P{\bar{A}}_{1,i} & \cdots & P{\bar{A}}_{m,i} \end{array}\right]\\ \Theta_{21,i}=\Theta_{12,i}^{T}\\ \Theta_{22,i}=\left[\begin{array}{ccc} B_{1,i}^{T}PB_{1,i}-Q_{1}+{\bar{\alpha }}_{1}I & 0 & 0\\ 0 & \ddots & 0\\ 0 & 0 & B_{m,i}^{T}PB_{m,i}-Q_{m}+{\bar{\alpha }}_{m}I \end{array}\right]\\ {\bar{\alpha }}_{k}=a_{k}^{2}\alpha_{k,1}+b_{k}^{2}(\alpha_{k,2}+\beta )+c_{k}^{2}\alpha_{k,3} \end{array}$$

*Therefore, the robust stabilization problem for the nonlinear stochastic time-delayed gene network in (22) becomes how to specify K*_*k*_, *k *= 0, 1,...,*m*, *in LMIs (27) such that there exist the positive definite symmetric common matrices P *> 0 *and Q*_*k *_> 0, *k *= 1,...,*m*, *i.e., the gene circuits K*_*k*_*g*_*k*_(*x*(*t *- *τ*_*k*_)), *k *= 0, 1,...,*m*, *could robustly stabilize the nonlinear stochastic time-delayed gene network, and the equilibrium point x *= 0 *of gene network (22) is asymptotically stable in probability under process delays and intrinsic molecular fluctuations*.

**Proof**. See Appendix D.   ■

**Remark 6 ***In the conventional fuzzy design *[33,34], *we need to design a complicated fuzzy controller or fuzzy filter for engineering systems. In this paper, the fuzzy approximation in (25) is only to simplify the gene circuit design procedure by solving K*_*k*_, *k *= 0, 1,...,*m*, *via LMIs in (27) instead of solving K*_*k*_, *k *= 0, 1,...,*m*, *via HJI directly. So there are some differences between the proposed fuzzy gene circuit design method and the conventional fuzzy control and filter design methods*.

**Remark 7 ***For the nonlinear stochastic gene network with time delays, it is diffcult to construct a Lyapunov function V *(*x*(*t*)) *to satisfy the HJI in (8) or (16). However, based on fuzzy approximation method, the nonlinear stochastic gene network with time delays is approximated by interpolating several local linear systems with time delays at different operation points. The advantage of the proposed fuzzy approach is that the circuit design procedure could be simplified from the linear time-delayed system design point of view. Thus the following standard Lyapunov function for linear system with multiple time-delays can be employed for the robust stabilization of nonlinear stochastic gene network *[32,35]

$$V(x(t))=x^{T}(t)Px(t)+\sum_{k=1}^{m}\int_{t-\tau_{k}}^{t}x^{T}(s)Q_{k}x(s)ds$$

*Therefore, we can avoid the diffculty of constructing a complex Lyapunov function for (16) and can employ the above Lyapunov function to obtain a systematic design procedure in the molecular circuit design*.

**Remark 8 ***If the gene circuit terms cannot be separated as (22) in gene circuit design and are merged into the terms f*_*k*_(*x*(*t *- *τ*_*k*_)), *k *= 0, 1,...,*m*, *as follows*

$$dx(t)=\sum_{k=0}^{m}f_{k}(x(t-\tau_{k}),K_{k})dt+\sum_{k=0}^{m}h_{k}(x(t-\tau_{k}))dW_{k}(t)$$

then (27) should be changed as follows

(28) $$\left[\begin{array}{ccc} \Xi_{11,i} & \Xi_{12,i} & \Xi_{13,i}\\ \Xi_{21,i} & -\Xi_{22,i} & 0\\ \Xi_{31,i} & 0 & -\Xi_{33,i} \end{array}\right]<0$$

*for all i *= 1,⋯,*L*, and *P *<*βI*, where

$$\begin{array}{l} \Theta_{11,i}=PA_{0,i}(K_{0})+A_{0,i}{\left(K_{0}\right)}^{T}P+{\bar{\alpha }}_{0}I+B_{0,i}^{T}PB_{0,i}+\sum_{k=1}^{m}Q_{k}\\ \Theta_{12,i}=\left[\begin{array}{ccc} PA_{1,i}(K_{1}) & \cdots & PA_{m,i}(K_{m}) \end{array}\right]\\ \Theta_{21,i}=\Theta_{12,i}^{T}\\ \Theta_{22,i}=\left[\begin{array}{ccc} B_{1,i}^{T}PB_{1,i}-Q_{1}+{\bar{\alpha }}_{1}I & 0 & 0\\ 0 & \ddots & 0\\ 0 & 0 & B_{m,i}^{T}PB_{m,i}-Q_{m}+{\bar{\alpha }}_{m}I \end{array}\right]\\ {\bar{\alpha }}_{k}=a_{k}^{2}\alpha_{k,1}+b_{k}^{2}(\alpha_{k,2}+\beta ) \end{array}$$

*and other matrices are same as in (27). A*_*k*,*i*_(*K*_*k*_), *k *= 0, 1,...,*m*, *denote the system matrices A*_*k*,*i*_, *k *= 0, 1,...,*m*, *containing the designed circuit parameters K*_*k*_, *k *= 0, 1,...,*m*, *as elements, respectively*.

After investigating the robust stabilization design of nonlinear stochastic gene network under process delays and intrinsic molecular fluctuations by the fuzzy approximation method, in order to avoid solving the nonlinear constrained optimization for molecular noise filtering problem in (17), the extrinsic molecular noise filtering ability improvement problem of gene network could be also treated by the fuzzy interpolation method. Therefore, the robust filtering circuit design problem becomes how to engineer the gene circuits *K*_*k*_*g*_*k*_(*x*(*t *- *τ*_*k*_)), *k *= 0, 1,..., *m*, not only to tolerate intrinsic molecular fluctuations $\sum_{k=0}^{m}h_{k}(x(t-\tau_{k}))dW_{k}(t)$ but also to attenuate the effect of extrinsic molecular noises *v*(*t*) on *z*_*o*_(*t*) to a prescribed noise filtering level *γ *as (14) or (15). Especially, when the prescribed noise filtering level *γ *is below the optimal filtering ability *γ*_0 _in (17) of the gene network, the robust filtering circuit design is needed to improve the molecular noise filtering ability. Since it is not easy to specify *K*_*k*_, *k *= 0, 1,...,*m*, to achieve robust filtering design directly for the nonlinear stochastic gene network in (22), the T-S fuzzy approximation in the following equation is employed to treat the robust filtering design problem as follows

(29) $$\begin{array}{c} dx(t)=\sum_{i=0}^{L}\mu_{i}(z)\left[\sum_{k=0}^{m}{\bar{A}}_{k,i}x(t-\tau_{k})dt+Gv(t)dt+\sum_{k=0}^{m}B_{k,i}x(t-\tau_{k})dW_{k}(t)\right]\\ +\sum_{k=0}^{k}\Delta f_{k}(x)dt+\sum_{k=0}^{m}K_{k}\Delta g_{k}(x)dt+\sum_{k=0}^{m}\Delta h_{k}(x)dW_{k}(t) \end{array}$$

(30) *z*_*o*_(*t*) = *Cx*(*t*)

Then we get the following result.

**Proposition 6 ***For the stochastic gene network with time delay in (29), if we could specify K*_*k*_, *i *= 1,...,*m for the designed gene circuits such that there exist the positive definite symmetric matrices P and Q*_*k*_, *k *= 1,...,*m, solving the following LMIs*

(31) $$\left[\begin{array}{ccccc} \Xi_{11,i}+\Phi & \Xi_{12,i} & \Xi_{13,i} & \Xi_{14,i} & \Xi_{15,i}\\ \Xi_{21,i} & -\Xi_{22,i} & 0 & 0 & 0\\ \Xi_{31,i} & 0 & -\Xi_{33,i} & 0 & 0\\ \Xi_{41,i} & 0 & 0 & -\Xi_{44,i} & 0\\ \Xi_{51,i} & 0 & 0 & 0 & -\gamma I \end{array}\right]<0$$

*for i *= 1, 2,⋯,*L, and P <βI where*

$$\begin{array}{ccc} \Phi =\left[\begin{array}{ccc} C^{T}C & 0 & \cdots \\ 0 & 0 & \cdots \\ \vdots & \vdots & \ddots \end{array}\right], & \Xi_{15,i}=\left[\begin{array}{c} PG\\ 0\\ \vdots \end{array}\right], & \Xi_{51,i}=\Xi_{15,i}^{T} \end{array}$$

*and the other matrices are defined in (27), then the effect of extrinsic molecular noises v(t) on the gene of *interest is less than *γ*.

**Proof**. See Appendix E.   ■

Therefore, according to fuzzy approximation method, the optimal *H*_∞ _noise filtering design problem for nonlinear stochastic time-delayed gene network in (29) could be solved by the following constrained optimization problem

(32) $$\begin{array}{l} \gamma_{0}=\underset{K_{k}}{\min}\gamma \\ \text{subject to }0<P<\beta I,\;Q_{k}>0\text{ and }(31). \end{array}$$

After solving *K*_*k*_, *k *= 0, 1,...,*m*, from LMIs in (31) for a prescribed noise filtering level *γ *or solving *K*_*k*_, *k *= 0, 1,...,*m*, from (32) for the optimal *H*_∞ _noise filtering ability *γ*_0_, we can design gene circuits *K*_*k*_*g*_*k*_(*x*(*t *- *τ*_*k*_)), *k *= 0, 1,...,*m*, in the nonlinear stochastic time-delayed gene network (22) to achieve a desired molecular noise filtering ability or an optimal *H*_∞ _noise filtering ability of gene network, respectively. Since the gene circuit design could improve the noise filtering ability, the γ_0_ in (32) should be less than the *γ*_*O *_in (17) without gene circuit design.

**Remark 9 ***In general, the optimization problem in (32) is called the eigenvalues problem, which can be efficiently solved by the Matlab LMI toolbox *[35].

**Remark 10 **If the gene control circuit terms cannot be separated as (22) and are merged into *f*_*k*_(*x*(*t *- *τ*_*k*_)), *k *= 0, 1,...,*m*, as follows

(33) $$\begin{array}{c} dx(t)=\sum_{k=0}^{m}f_{k}(x(t-\tau ),K_{k})dt+Gv(t)dt+\sum_{k=0}^{m}h_{k}(x(t-\tau_{k}))dW_{k}(t) \end{array}$$

(34) *z*_*o*_(*t*) = *Cx*(*t*)

then LMIs in (31) should be modified as follows

(35) $$\left[\begin{array}{cccc} \Xi_{11,i}+\Phi & \Xi_{12,i} & \Xi_{13,i} & \Xi_{15,i}\\ \Xi_{21,i} & -\Xi_{22,i} & 0 & 0\\ \Xi_{31,i} & 0 & -\Xi_{33,i} & 0\\ \Xi_{51,i} & 0 & 0 & -\gamma I \end{array}\right]<0$$

for i = 1, 2,⋯,L, and P <βI, where

$$\begin{array}{l} \Theta_{11,i}=PA_{0,i}(K_{0})+A_{0,i}{\left(K_{0}\right)}^{T}P+{\bar{\alpha }}_{0}I+B_{0,i}^{T}PB_{0,i}+\sum_{k=1}^{m}Q_{k}\\ \Theta_{12,i}=\left[\begin{array}{ccc} PA_{1,i}(K_{1}) & \cdots & PA_{m,i}(K_{m}) \end{array}\right]\\ \Theta_{21,i}=\Theta_{12,i}^{T}\\ \Theta_{22,i}=\left[\begin{array}{ccc} B_{1,i}^{T}PB_{1,i}-Q_{1}+{\bar{\alpha }}_{1}I & 0 & 0\\ 0 & \ddots & 0\\ 0 & 0 & B_{m,i}^{T}PB_{m,i}-Q_{m}+{\bar{\alpha }}_{m}I \end{array}\right]\\ {\bar{\alpha }}_{k}=a_{k}^{2}\alpha_{k,1}+b_{k}^{2}(\alpha_{k,2}+\beta ) \end{array}$$

*and other matrices are same as in (27) and (31). A_*k*,*i*_(K_*k*_), k = 0, 1,...,m, denote the matrices A_*k*,*i *_whose entries contain circuit kinetic parameters K_*k*_. In this situation, for a prescribed noise filtering ability γ, the robust circuit design is to specify K_*k*_, k = 0, 1,...,m, such that there exist P > 0 and Q_*k *_> 0 for LMIs in (35)*.

**Remark 11 ***If the prescribed attenuation level γ is small in H_∞ _noise filtering design, more extrinsic molecular noises are eliminated by gene network. However, it is more diffcult to solve LMIs in (31) or (35) and more control or filtering effort is needed. This is a trade off for a designer between a good filtering ability (small γ*) and a design diffculty in the robust gene circuit design.

**Remark 12 ***Unlike the conventional fuzzy control designs *[30,33,34,47]* and fuzzy filter designs *[20,27,28], *the proposed fuzzy interpolation method is only to simplify the circuit design procedure so that we could solve K*_*k*_, *k *= 0, 1,...,*m, easier via the LMI scheme*.

According to the above analysis, a robust circuit design procedure using fuzzy interpolation method for stochastic gene regulatory networks are summarized as follows

### Design Procedure

**Step 1: **Model the nonlinear time-delayed gene network as (12) and shift the interested equilibrium point to origin as (18).

**Step 2: **Design some feasible feedback control circuits *K*_0_*g*_0_(*x*(*t*)) and *K*_*k*_*g*_*k*_(*x*(*t *- *τ*_*k*_)), *k *= 1,...,*m*.

**Step 3: **Approximate the nonlinear stochastic gene network by fuzzy system as (29).

**Step 4: **Estimate the fuzzy approximation error bounds *a*_*k*_, *b*_*k*_, and *c*_*k*_, *k *= 0,1,...,*m*, in (26), and give a prescribed noise filtering ability *γ*.

**Step 5: **Specify the feasible circuit kinetic parameters *K*_*k*_, *k *= 0,1,...,*m*, by solving LMIs in (31) for the prescribed noise filtering ability *γ *or solving the constrained optimization in (32) for the optimal noise filtering ability *γ*_0_.

### Computational Design Examples in Silico

Two computational design examples using the developed robust gene circuit design method are presented in silico to illustrate the design procedure and to validate the performance of the proposed molecular circuit design methods.

**Figure 2:**
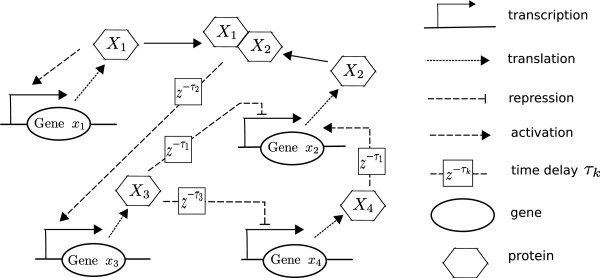
**Gene regulatory network comprising four genes***** x***_**1**_**-*****x***_**4**_[**36**].

Example 1:

Consider a benchmark genetic regulatory network as shown in Fig. 2, which is a typical gene regulatory network describing the gene, mRNA and protein interactions [36]. These genes are regulated by other genes and then expressed through transcription and translation to obtain their products, i.e. proteins. Then these proteins could be as the TFs of other genes to regulate the expressions of other genes after some process delays. We consider only the mRNA abundances *x*_1_, *x*_2_, *x*_3_, and *x*_4_. Suppose the gene network suffers from stochastic intrinsic molecular fluctuations and extrinsic molecular noises v(t) on gene *x*_2 _and gene *x*_4_. The stochastic gene network under process delays, intrinsic molecular fluctuations and extrinsic molecular noises can be represented as follows [36]

(36) *dx*_1_(*t*) = (1 - *λ*_1_*x*_1_(*t*))*dt*

(37) $$\begin{array}{c} dx_{2}(t)=\left(\frac{V_{2}x_{4}^{n_{4}}(t-\tau_{1})}{\left(\Lambda_{2}+x_{4}^{n_{4}}(t-\tau_{1}))(\Lambda_{I3}+x_{3}^{n_{3}}(t-\tau_{1})\right)}\right)\\ -\lambda_{2}x_{2}(t)+0.25v(t))dt-0.1x_{2}(t)dW_{0}(t) \end{array}$$

(38) $$dx_{3}(t)=\left(\frac{V_{3}{\left(x_{1}(t-\tau_{2})x_{2}(t-\tau_{2})\right)}^{n_{12}}}{\Lambda_{3}+{\left(x_{1}(t-\tau_{2})x_{2}(t-\tau_{2})\right)}^{n_{12}}}-\lambda_{3}x_{2}(t)\right)dt$$

(39) $$\begin{array}{c} dx_{4}=\left(\frac{V_{4}}{\Lambda_{I3}+x_{3}^{n_{3}}(t-\tau_{3})}-\lambda_{4}x_{4}(t)+0.25v(t)\right)dt\\ +0.7x_{3}(t-\tau_{3})dW_{2}(t) \end{array}$$

Here *λ*_1_, *λ*_2_, *λ*_3 _and *λ*_4 _are the first-order rate constants of the degradation of *x*_1_, *x*_2_, *x*_3 _and *x*_4_, respectively. *τ*_*k *_denotes the process delay of gene regulation due to transcription, transfection, posttranslation modification and transportation of TF. The Hill term $\frac{V_{2}x_{4}^{n_{4}}(t-\tau_{1})}{\left(\Lambda_{2}+x_{4}^{n_{4}}(t-\tau_{1})\right)}$ describes the sigmoid formation of *x*_2 _activated by *x*_4 _with time delay *τ*_1_, maximal rate *V*_2_, dissociation constant Λ_2 _and Hill coefficient *n*_4_. The inhibition by *x*_3 _is expressed by the term (Λ_*I*3 _+ $x_{3}^{n_{3}}$(*t *- *τ*_3_)). The formation of *x*_3 _is modeled with Hill expression that points to a threshold of the formation of *x*_3 _depending on the concentrations of *x*_1 _and *x*_2_. *V*_3 _and Λ_3 _are maximal rate and dissociation constant, respectively, and *n*_12 _is the Hill coefficient. The production of *x*_4 _depends on the maximal rate *V*_4 _and on the inhibition by *x*_3_. The parameters are chosen as follows [36]

**Figure 3:**
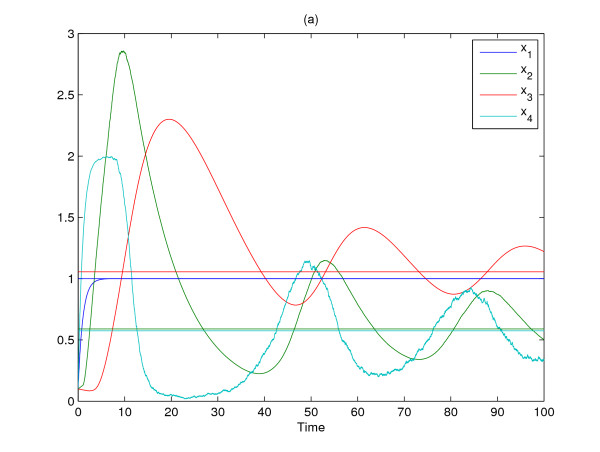
**The gene expression profiles of nonlinear stochastic time-delayed gene network without engineered gene circuit**.

*λ*_1 _= 1; *V*_2 _= 1; Λ_2 _= 5; *λ*_2 _= 0.1; Λ_*I*3 _= 0.5;

*n*_3 _= 1; *V*_3 _= 1; Λ_3 _= 5; *λ*_3 _= 0.1; *V*_4 _= 1; *λ*_4 _= 1;

*n*_4 _= 1; *n*_12 _= 1; *τ*_1 _= 1; *τ*_2 _= *τ*_3 _= 2;

The gene expression profiles of stochastic gene network with process delays, intrinsic and extrinsic molecular noises in (36)–(39) are given in Fig. 3. It is seen that there are large fluctuations in these gene expression profiles due to process delays and molecular noises. Suppose the proposed gene circuit design method is employed to attenuate these molecular fluctuations to achieve a desired steady state quickly. In general, engineering a gene circuit to a gene network may change its steady state because of nonlinear inherence. Since changing the steady state of a gene network may destroy the normal function of the gene network, it is important to make sure that a designed gene circuit does not change the steady state (phenotype) of a gene network while it can compensate process delays and attenuate molecular noises. Therefore, in order to keep the steady state of a designed gene network the same as the desired steady state, the gene network is designed as follows

(40) $$dx_{1}=(1-\lambda_{1}x_{1}(t))dt$$

(41) $$\begin{array}{c} dx_{2}(t)=(\frac{k_{1}V_{2}x_{4}^{n4}(t-\tau_{1})}{\left(\Lambda_{2}+x_{4}^{n4}(t-\tau_{1}))(\Lambda_{I3}+x_{3}^{n3}(t-\tau_{1})\right)}\\ -k_{1}\lambda_{2}x_{2}(t)+0.25v(t))dt-0.1x_{2}(t)dW_{0}(t) \end{array}$$

(42) $$dx_{3}(t)=\left(\frac{k_{2}V_{3}{\left(x_{1}(t-\tau_{2})x_{2}(t-\tau_{2})\right)}^{n_{12}}}{\Lambda_{3}+{\left(x_{1}(t-\tau_{2})x_{2}(t-\tau_{2})\right)}^{n_{12}}}-k_{2}\lambda_{3}x_{3}(t)\right)dt$$

(43) $$\begin{array}{c} dx_{4}(t)=\left(\frac{V_{4}}{\Lambda_{I3}+x_{3}{\left(t-\tau_{3}\right)}^{n_{3}}}-\lambda_{4}x_{4}(t)+0.25v(t)\right)dt\\ +0.7x_{3}(t-\tau_{3})dW_{2}(t) \end{array}$$

where *k*_1_, *k*_2 _are the circuit kinetic parameters at the genes *x*_2 _and *x*_3_. From the simulation result of the nominal gene regulatory network, we can know that the equilibrium point of the nominal system is at [*x*_*e*1_, *x*_*e*2_, *x*_*e*3_, *x*_*e*4_] = [1.0000, 0.5899, 1.0557, 0.5739] which is the desired steady state (phenotype) and should be shifted to the origin as shown in (18) in the design procedure. Suppose we want to specify the engineered circuit parameters *k*_1 _and *k*_2 _such that the prescribed noise filtering ability *γ *= 0.9 can be achieved for the molecular noise filtering of perturbative gene regulatory network in (40)–(43). We must find the operative points of the gene network to construct the fuzzy model at first. The operative points of the state *x*_1 _are located at (0.1, 1). For the other states *x*_2_, *x*_3_, and *x*_4_, the operative points are located at (0, 1), (0.5, 1.5), and (0, 1), respectively. Then we can create two membership functions for every state at the operative points, and the number of fuzzy rules is *L *= 16. The bounds of the fuzzy approximation errors are estimated as *a*_0 _= 0, *a*_1 _= 0.001, *a*_2 _= 0.0524, *b*_0 _= 0, *b*_1 _= 0, *b*_2 _= 0, *c*_0 _= 0, *c*_1 _= 0, *c*_2 _= 0 at (26). By the robust molecular noise filtering circuit design in Proposition 4, in order to guarantee the positive definite symmetric matrices of *P*, *Q*_1 _and *Q*_2_, the engineered circuit kinetic parameters *k*_1 _and *k*_2 _should be in the range *k*_1 _∈ (0, 1] and *k*_2 _∈ [1, 20]. In the case *k*_1 _= 0.1, *k*_2 _= 10, we get

$$\begin{array}{c} P=\left[\begin{array}{llll} 8.4244 & 0.0001 & -0.0001 & -0.0004\\ 0.0001 & 0.4647 & 0.0003 & -0.0002\\ -0.0001 & 0.0003 & 0.0227 & 0.0006\\ -0.0004 & -0.0002 & 0.0006 & 0.0283 \end{array}\right],\\ Q_{1}=\left[\begin{array}{llll} 2.8725 & -0.0007 & 0.0053 & -0.0039\\ -0.0007 & 0.0010 & 0.0026 & -0.0010\\ 0.0053 & 0.0026 & 0.0659 & -0.0096\\ -0.0039 & -0.0010 & -0.0096 & 0.0162 \end{array}\right],Q_{2}=\left[\begin{array}{llll} 2.8978 & 0.0032 & 0.0067 & 0.0028\\ 0.0032 & 0.0324 & 0.0016 & 0.0008\\ 0.0067 & 0.0016 & 0.1328 & 0.0096\\ 0.0028 & 0.0008 & 0.0096 & 0.0304 \end{array}\right] \end{array}$$

**Figure 4:**
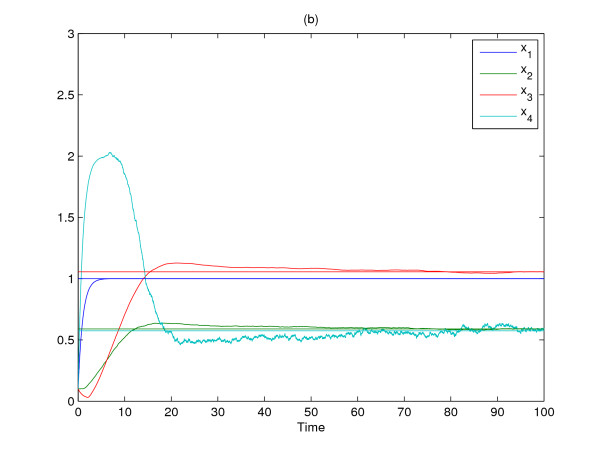
**The gene expression profiles of nonlinear stochastic time-delayed gene network with engineered gene circuit**. Four step functions in figures denote the desired steady states of gene expressions *x*_1_-*x*_4_.

The molecular noise filtering results without engineered circuit and with two engineered circuit *k*_1 _= 0.1 and *k*_2 _= 10 are simulated in Fig. 3 and Fig. 4, respectively. Obviously, the large molecular fluctuations in Fig. 3 are significantly attenuated by the proposed gene circuits as shown in Fig. 4 and the expression profiles of four genes asymptotically achieve the desired states in probability. From the simulation, the gene network without engineered gene circuit design has much molecular fluctuations due to process delays and molecular noises. However, the designed gene network by the proposed gene circuit design can robustly achieve the desired equilibrium point *x*_*e *_= [1.0000, 0.5899, 1.0557, 0.5739] and the molecular noise filtering is also improved by the proposed gene circuit design. For confirmation, the noise filtering ability of the designed gene network is estimated as follows

$$\frac{{\left(E\int_{0}^{100}z_{o}^{T}(t)z_{o}(t)dt\right)}^{1/2}}{{\left(E\int_{0}^{100}v^{T}(t)v(t)dt\right)}^{1/2}}\approx 0.3475<\gamma =0.9$$

and the filtering ability of stochastic gene network in (36)–(39) without gene circuit design is given by

$$\frac{{\left(E\int_{0}^{100}z_{o}^{T}(t)z_{o}(t)dt\right)}^{1/2}}{{\left(E\int_{0}^{100}v^{T}(t)v(t)dt\right)}^{1/2}}\approx 6.2545>\gamma =0.9$$

where *z*_*o*_(*t*) = *Cx*(*t*) in which *C *is an identity matrix. Therefore, the prescribed filtering ability *γ *is achieved by the proposed robust gene circuit design. From the design example in silico, it is obvious that the proposed gene circuit design could improve the molecular noise filtering ability of the nonlinear stochastic gene network.

Recently, the gene circuit design can be implemented by using a highly efficient phage-based homologous recombination system, called recombineering [37,38]. This powerful technology has been used to engineer large segments of genomic DNA to generate transgenic and knockout construct to insert or delete the TF binding sites in the promoter region of regulated genes to increase or decrease the expression of regulated genes. Therefore, *k*_1 _= 0.1 of the first term in the right hand side of (41) could be achieved by deleting 90% of TF binding sites of gene product *X*_4 _of gene *x*_4 _from the promoter region of gene *x*_2 _through knockout construct technique of recombineering. Similarly, *k*_2 _= 10 of first term in the right hand side of (42) could be implemented by inserting 10 times of TF binding sites of complex protein *X*_1_*X*_2 _of gene *x*_1 _and *x*_2 _to the promoter region of gene *x*_3 _to increase its gene expression.

As for the implementation of two mRNA decay terms *k*_1_*λ*_2_*x*_2 _and *k*_2_*λ*_3_*x*_3 _in (41) and (42), respectively, it has been shown that mRNA decay in eukaryotic cells can be achieved by shortening the 3' polyadenylate tail found on eukaryotic mRNAs (referred to as deadenylation), which primarily triggers decapping, leading to 5' to 3' exonucleolysis. Alternatively, removal of 3' polyadenylate tail can expose the mRNA to 3' to 5' degradation [39,40,44-46]. Therefore, by elongating the 3' polyadenylate tail of mRNA of gene *x*_2_, we can get a small kinetic decay parameter *k*_1 _of *k*_1_*λ*_2_*x*_2 _in (41) and by shortening the 3' polyadenylate tail of gene *x*_3_, we can get a large kinetic decay parameter *k*_2 _of *k*_2_*λ*_3_*x*_3 _in (42).

**Figure 5:**
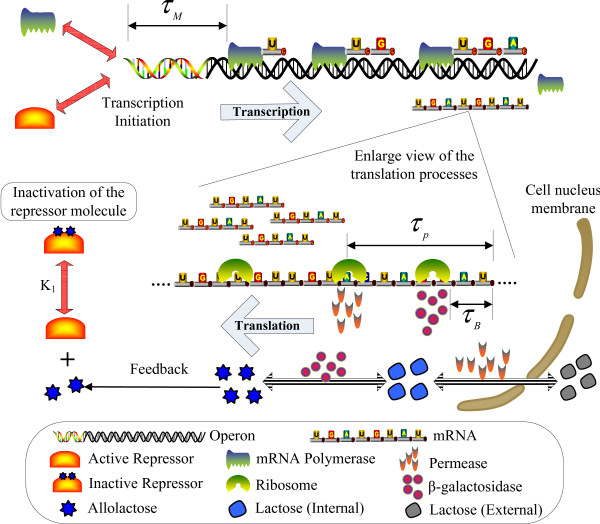
**The schematic diagram of the lactose operon regulatory system**.

Example 2:

Consider a dynamic system for the regulation of induction in the *lac *operon [48]. This stochastic system consists of five nonlinear differential equations with multiple time delays due to transcription and translation processes (see Fig. 5). Therefore, this gene regulatory system in vivo could be described by the following nonlinear stochastic time-delayed system [48].

(44) $$dx_{1}(t)=\left(\alpha_{M}\frac{1+K_{1}{\left(\delta_{M}x_{3}(t-\tau_{M})\right)}^{2}}{K_{2}+K_{1}{\left(\delta_{M}x_{3}(t-\tau_{M})\right)}^{2}}+\Gamma_{0}-(\gamma_{M}+\mu )x_{1}(t)\right)dt$$

(45) $$dx_{2}(t)=(\alpha_{B}\delta_{B}x_{1}(t-\tau_{B})-(\gamma_{B}+\mu )x_{2}(t))\;dt$$

(46) $$\begin{array}{c} dx_{3}(t)=\left(\alpha_{A}x_{2}(t)\frac{x_{4}(t)}{K_{L}+x_{4}(t)}-\beta_{A}x_{2}(t)\frac{x_{3}(t)}{K_{A}+x_{3}(t)}-(\gamma_{A}+\mu )x_{3}(t)\right)dt\\ +(\Delta_{1}x_{2}(t)x_{4}(t)-\Delta_{2}x_{2}(t)x_{3}(t))dW_{0} \end{array}$$

(47) $$\begin{array}{c} dx_{4}(t)=\left(\alpha_{L}x_{5}(t)\frac{L_{e}}{K_{L_{e}}+L_{e}}-\beta_{L_{1}}x_{5}(t)\frac{x_{4}(t)}{K_{L_{1}}+x_{4}(t)}-\beta_{L_{2}}x_{2}(t)\frac{x_{4}(t)}{K_{L_{2}}+x_{4}(t)}-(\gamma_{L}+\mu )x_{4}(t)\right)dt\\ +(\Delta_{3}-\Delta_{4}x_{4}(t)+\Delta_{5}x_{5}(t)x_{4}(t))dW_{0}+0.15v(t) \end{array}$$

(48) $$dx_{5}(t)=(\alpha_{P}\delta_{P}x_{1}(t-\tau_{P})-(\gamma_{P}+\mu )x_{5}(t))dt$$

where *x*_1_(*t*) is the dynamic of mRNA production; *x*_2_(*t*) is the dynamic of *β*-galactosidase; the dynamic of allolactose is described by *x*_3_(*t*); the lactose dynamic is described by *x*_4_(*t*); the permease dynamic is described by *x*_5_(*t*). When the the glucose available for cellular metabolism is absent, the external lactose *L*_*e *_is transported to the cell by the permease *x*_5_(*t*). Then, by the enzyme *β*-galactosidase *x*_2_(*t*), the intracellular lactose *x*_4_(*t*) is broken down into glucose, galactose, and allolactose *x*_3_(*t*). Finally, the allolactose feeds back to bind with the lactose repressor and enables the transcription process to produce the mRNA production *x*_1_(*t*). The mRNA production *x*_1_(*t*) from DNA via transcription needs a delayed time *τ*_*M *_for RNA polymerase to transverse the three structural genes, and the *β*-galactosidase production *x*_2_(*t*) through mRNA translation requires a delayed time *τ*_*B*_. The delayed time *τ*_*P *_is the translation time between mRNA and permease. The delay *τ*_*P *_is the sum of the *β*-galactosidase and premise translation times based on the assumption that permease production can not start until *β*-galactosidase production is complete. Δ_*i*_, *i *= 1,...,5 denote the corresponding parameter variations. The detailed process for the lactose operon regulatory system is described in Fig. 5 and refers to the literature [48]. The parameters for the model are given in Table 1. The initial values are chosen as *x*_1_(0) = 6.26 × 10^-4^, *x*_2_(0) = 0, *x*_3_(0) = 3.80 × 10^-1^, *x*_4_(0) = 3.72 × 10^-1^, and *x*_5_(0) = 1.49 × 10^-2^. The desired steady state (the interested equilibrium point) is at *x*_*e*_(*t*) = [1.2359 × 10^-3^, 8.3645 × 10^-4^, 5.7666 × 10^-1^, 4.1583 × 10^-1^, 1.1664 × 10^-2^]^*T*^.

**Table 1:** The parameters for the lactose operon regulatory system.

*Le*	8.50 × 10^-2 ^mM	*μ*	2.26 × 10^-2 ^min^-1^
*γ*_*M*_	0.411 min^-1^	*γ*_*B*_	8.33 × 10^-4 ^min^-1^
*γ*_*A*_	0.52 min^-1^	*γ*_*L*_	1.6043 min^-1^
*γ*_*P*_	0.65 min^-1^	Γ_0_	7.25 × 10^-7 ^mM/min
*α*_*M*_	9.97 × 10^-4 ^mM/min	*α*_*B*_	1.66 × 10^-2 ^min^-1^
*α*_*A*_	1.76 × 10^4 ^min^-1^	*α*_*L*_	2908.8 min^-1^
*α*_*P*_	10.0 min^-1^	*β*_*A*_	2.15 × 10^4 ^min^-1^
	2.65 × 10^3 ^min^-1^	$\beta_{L_{2}}$	7.614 × 10^3 ^min^-1^
*K*_1_	2.52 × 10^4 ^(mM)^-2^	*K*_2_	7200
*K*_*A*_	1.95 mM	*K*_*L*_	9.7 × 10^-1 ^mM
$K_{L_{e}}$	0.26 mM	$K_{L_{1}}$	1.81 mM
$K_{L_{2}}$	9.72 × 10^-1^mM	*τ*_*M*_	0.1 min
*τ*_*B*_	2.0 min	*τ*_*P*_	2.83 min
*δ*_*M*_	9.9774 × 10^-1^	*δ*_*B*_	9.5580 × 10^-1^
*δ*_*P*_	9.3804 × 10^-1^	Δ_1_	2.5
Δ_2_	1.803	Δ_3_	2.079
Δ_*4*_	5	Δ_5_	1

**Figure 6:**
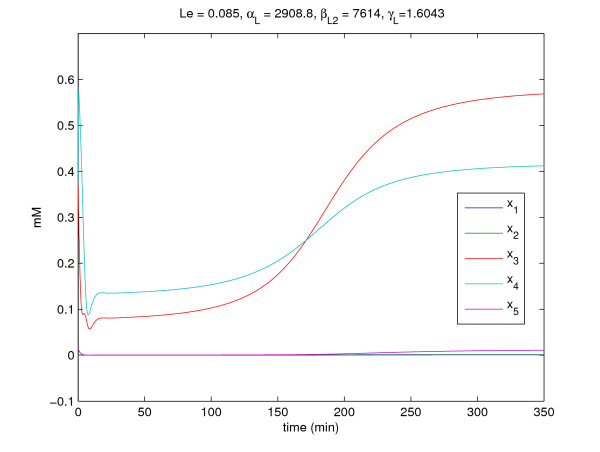
**The time-profiles of the lactose operon regulatory system in example 2 which is free of the intrinsic molecular fluctuations and extrinsic disturbances**.

**Figure 7:**
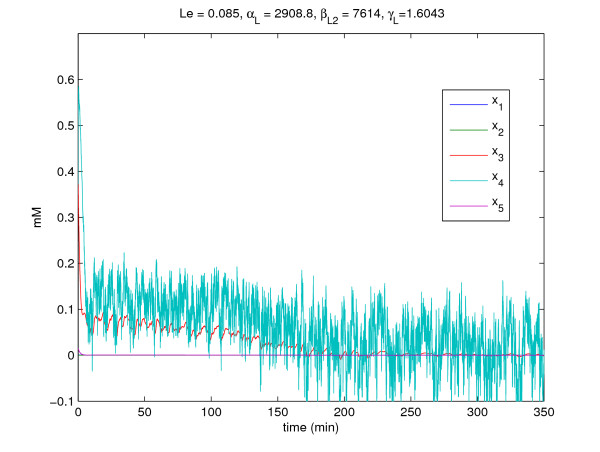
**The time-profiles of the stochastic lactose operon regulatory system in example 2 without circuit design**.

If the gene regulatory system is free of intrinsic molecular fluctuations and extrinsic disturbances, i.e., *W*(*t*) ≡ 0 and *v*(*t*) ≡ 0, the time-profiles of regulatory system are shown in Fig. 6. We can know the system can work right. However, in the realistic cellular environment, this gene regulatory system may suffer from the intrinsic molecular fluctuations and extrinsic disturbances. In this example, an extrinsic disturbance *v*(*t*) affects the lactose dynamic *x*_4_(*t*), and the allolactose dynamic *x*_3_(*t*) and the lactose dynamic *x*_4_(*t*) also suffer from some molecular fluctuations. By computational simulation, the time profiles of the stochastic regulatory system are shown in Fig. 7. Obviously, this system can not work properly. Therefore, we need to redesign the some parameters of some gene circuits to ensure the system can resist the influences of intrinsic molecular fluctuations and extrinsic disturbances. From the lactose dynamic *x*_4_(*t*) which is critically destroyed, we choose parameters *L*_*e*_, *α*_*L*_, $\beta_{L_{2}}$, and *γ*_*L *_to redesign the gene regulatory system.

By the same procedure as example 1, the we could choose the three membership functions for every state *x*_*i*_(*t*) to approximate nonlinear functions of the gene network. Therefore, we have *L *= 3^5 ^= 243 fuzzy rules to approximate the gene regulatory system. The approximation errors in (26) could be estimated as *a*_0 _= 4.2355 × 10^-3^, *a*_1 _= 1.2136 × 10^-9^, *a*_2 _= 4.8344 × 10^-4^, *a*_3 _= 7.7390 × 10^-17^, *b*_0 _= 2.7578 × 10^-7^, *b*_1 _= 0, *b*_2 _= 0, and *b*_3 _= 0. Note that the parameters of the fuzzy system are omitted, because the amount of the parameters is huge. Using LMI toolbox in Matlab to solve the optimization problem in (32), we can obtain the optimal *H*_∞ _filtering ability *γ*_0 _= 0.3535 and the positive definite matrices *P*, *Q*_*k*_, *k *= 1...3 in the following.

$$P=\left[\begin{array}{lllll} 2.5408\times 10^{-1} & 4.8426\times 10^{-20} & -2.1695\times 10^{-19} & -5.4464\times 10^{-18} & -7.5890\times 10^{-21}\\ 4.8426\times 10^{-20} & 2.4970\times 10^{-1} & 4.7942\times 10^{-19} & -4.3807\times 10^{-3} & 5.0867\times 10^{-20}\\ -2.1695\times 10^{-19} & 4.7942\times 10^{-19} & 2.5408\times 10^{-1} & 1.1042\times 10^{-17} & -5.0226\times 10^{-18}\\ -5.4464\times 10^{-18} & -4.3807\times 10^{-3} & 1.1042\times 10^{-17} & 2.4970\times 10^{-1} & -3.2363\times 10^{-18}\\ -7.5890\times 10^{-21} & 5.0867\times 10^{-20} & -5.0226\times 10^{-18} & -3.2363\times 10^{-18} & 2.54083\times 10^{-1} \end{array}\right]$$

$$Q_{1}=\left[\begin{array}{ccccc} 1.0671 & 7.3044\times 10^{-19} & -3.5261\times 10^{-20} & -4.4442\times 10^{-18} & 3.7035\times 10^{-20}\\ 7.3044\times 10^{-19} & 1.0671 & 1.9971\times 10^{-18} & -2.2293\times 10^{-17} & 3.6867\times 10^{-19}\\ -3.5261\times 10^{-20} & 1.9971\times 10^{-18} & 1.0671 & 2.4345\times 10^{-16} & -5.5866\times 10^{-18}\\ -4.4442\times 10^{-18} & -2.2293\times 10^{-17} & 2.4345\times 10^{-16} & 1.0671 & -2.3426\times 10^{-18}\\ 3.7035\times 10^{-20} & 3.6867\times 10^{-19} & -5.5866\times 10^{-18} & -2.3426\times 10^{-18} & 1.0671 \end{array}\right]$$

$$Q_{2}=\left[\begin{array}{ccccc} 1.4228 & -1.2435\times 10^{-18} & 9.3471\times 10^{-19} & -1.4067\times 10^{-18} & -3.3703\times 10^{-20}\\ -1.2435\times 10^{-18} & 1.4228 & 1.7898\times 10^{-18} & 1.6799\times 10^{-17} & 5.8180\times 10^{-20}\\ 9.3471\times 10^{-19} & 1.7898\times 10^{-18} & 1.4228 & -1.5079\times 10^{-16} & 1.7036\times 10^{-18}\\ -1.4067\times 10^{-18} & 1.6799\times 10^{-17} & -1.5079\times 10^{-16} & 1.4228 & 5.7594\times 10^{-18}\\ -3.3703\times 10^{-20} & 5.8180\times 10^{-20} & 1.7036\times 10^{-18} & 5.7594\times 10^{-18} & 1.4228 \end{array}\right]$$

$$Q_{3}=\left[\begin{array}{ccccc} 4.2786 & 2.4871\times 10^{-4} & -1.0347\times 10^{-2} & -1.6371\times 10^{-2} & -7.7195\times 10^{-5}\\ 2.4871\times 10^{-4} & 4.2808 & -8.8741\times 10^{-3} & -6.9607\times 10^{-2} & -1.6886\times 10^{-4}\\ -1.0347\times 10^{-2} & -8.8741\times 10^{-3} & 5.8106 & 9.6102\times 10^{-1} & 2.2939\times 10^{-2}\\ -1.6371\times 10^{-2} & -6.9607\times 10^{-2} & 9.6102\times 10^{-1} & 4.9602 & 1.5395\times 10^{-2}\\ -7.7195\times 10^{-5} & -1.6886\times 10^{-4} & 2.2939\times 10^{-2} & 1.5395\times 10^{-2} & 4.2788 \end{array}\right]$$

The design parameters of this regulation system are obtained as *L*_*e *_= 8.0 × 10^-2^, *α*_*L *_= 2880.3, $\beta_{L_{2}}$ = 8460, and *γ*_*L *_= 0.002.

**Figure 8:**
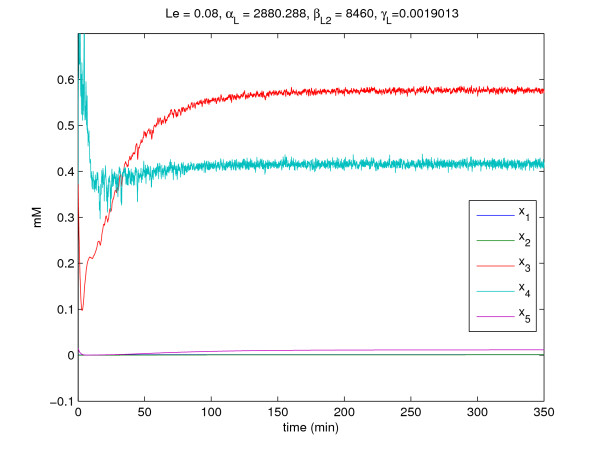
**The time-profiles of the stochastic lactose operon regulatory system in example 2 with circuit design**.

From the simulation results in Fig. 7 and Fig. 8, the molecular noise filtering ability is also improved significantly by the proposed gene circuit design. For confirmation, the filtering ability of stochastic regulatory systems of *lac *operon without gene circuit design is given by

$$\frac{{\left(E\int_{0}^{100}z_{o}^{T}(t)z_{o}(t)dt\right)}^{1/2}}{{\left(E\int_{0}^{100}v^{T}(t)v(t)dt\right)}^{1/2}}\approx 6.8949>\gamma_{0}=0.3535$$

and the noise filtering ability of the designed regulatory system is estimated as follows

$$\frac{{\left(E\int_{0}^{100}z_{o}^{T}(t)z_{o}(t)dt\right)}^{1/2}}{{\left(E\int_{0}^{100}v^{T}(t)v(t)dt\right)}^{1/2}}\approx 0.10525<\gamma_{0}=0.3535$$

The conservative result of noise filtering ability is mainly due to the comservative procedure in solving LMIs [35].

## Conclusion

In the study, a gene network with process delays, intrinsic molecular fluctuations and extrinsic molecular noises is modeled as a nonlinear stochastic time-delayed system. Then we propose a stochastic gene circuit design method for the improvement of robust stability and molecular noise filtering ability of nonlinear gene network to tolerate time-delays and to attenuate molecular noises via LMI technique and fuzzy interpolation scheme. The T-S fuzzy system can approach the nonlinear stochastic gene network with time delays via the interpolation of several local linear time-delayed systems to avoid solving HJI in nonlinear robust stabilization and filtering design problems. Therefore, the robust circuit design procedure for the nonlinear stochastic time-delay gene network can be simplified by specifying circuit kinetic parameters to satisfy a set of LMIs, which could be efficiently solved by the LMI toolbox in Matlab. Unlike the conventional trial-and-error, the proposed design method provides a systematic method for robust gene circuit design of nonlinear stochastic time-delayed gene networks. Because the microarray data become popular, the construction of a dynamic model from microarray data for a gene regulatory network becomes possible. Furthermore, the experimental advances in transfection and transformation biotechnologies make gene circuit implementation easier. Therefore, the proposed gene circuit design methods have much potential for application to systems biology, synthetic biology and drug design when a gene regulatory network has to be designed for improving its robust stability and filtering ability of disease-perturbed gene networks or when a synthetic gene network needs to perform reliably around a desired equilibrium point despite of process delays, intrinsic molecular fluctuations and extrinsic molecular noises in host cell. Finally, a benchmark design example is also given in silico to illustrate the design procedure and to validate the proposed robust circuit design method in nonlinear stochastic time-delayed gene regulatory networks.

In the future, we will focus on the development of some more general design methods for robust synthetic biologic networks under parameter fluctuations, time-delays and environmental molecular noise in host cell. After some design specifications are given beforehand, for example, the magnitudes of parameter variations to be tolerated, the possible delays to be compensated, the filtering ability to attenuate the molecular noises, the feasible ranges of kinetic parameters to be designed and the desired steady states to be achieved, we want to develop some systematic design methods for a synthetic biologic network to meet these design specifications and achieve the design objective.

## Appendix

Before the proof of propositions, the following fact is necessary.

**Fact 1 **([35]):

$$X^{T}PY+Y^{T}PX\leq \frac{1}{\alpha }X^{T}PX+\alpha Y^{T}PY$$

for vectors *X*, *Y*, a constant *α *> 0 and a positive definite matrix *P *> 0 with appropriate dimensions.

### **Appendix A **Proof of Proposition 1

By the stochastic Lyapunov stability theorem, we choose a Lyapunov function *V*(*x*(*t*)) > 0 such that $E\frac{d}{dt}V(x(t))<0$. Using Itô formula [49], we have

(A.1) $$\begin{array}{c} dV(x(t))={\left(\frac{\partial V(x(t))}{\partial x}\right)}^{T}\left(\sum_{k=0}^{m}f_{k}(x(t-\tau_{k}))dt+\sum_{k=0}^{m}h_{k}(x(t-\tau_{k}))dW_{k}(t)\right)\\ +\sum_{k=0}^{m}\frac{1}{2}h_{k}^{T}(x(t-\tau_{k}))\frac{\partial^{2}V(x(t))}{\partial x^{2}}h_{k}(x(t-\tau_{k}))dt \end{array}$$

Then

(A.2) EddtV(x(t))=E{(∂V(x(t))∂x)T(∑k=0mfk(x(t−τk)))+∑k=0m12hkT(x(t−τk))∂2V(x(t))∂x2hk(x(t−τk))}

If the inequality in (8) holds, then $E\frac{d}{dt}V(x(t))<0$, i.e., the nonlinear stochastic gene network in (7) is asymptotically stable in probability at *x *= 0.

### **Appendix B **Proof of Proposition 2

For the linear stochastic gene network in (6), if we choose

$$V(x(t))=x^{T}(t)Px(t)+\sum_{k=1}^{m}\int_{t-\tau_{k}}^{t}x^{T}(s)Q_{k}x(s)ds$$

where *P *= *P*^*T *^> 0 and $Q_{k}=Q_{k}^{T}>0$, then

(B.1) $$\begin{array}{c} E\frac{d}{dt}V(x(t))=E\{x^{T}(t)P\sum_{k=1}^{m}A_{k}x(t-\tau_{k})+(\sum_{k=1}^{m}A_{k}x(t-\tau_{k}){)}^{T}Px(t)+\sum_{k=0}^{m}x^{T}(t-\tau_{k})B_{k}^{T}P\;B_{k}x(t-\tau_{k})\\ +\sum_{k=1}^{m}\left(x^{T}(t)Q_{k}x(t)-x^{T}(t-\tau_{k})Q_{k}x(t-\tau_{k})\right)\} \end{array}$$

(B.2) $$=E{\bar{x}}^{T}\left[\begin{array}{cccc} \Xi & PA_{1} & \cdots & PA_{m}\\ A_{1}^{T}P & B_{1}^{T}PB_{1}-Q_{1} & 0 & 0\\ \vdots & 0 & \ddots & 0\\ A_{m}^{T}P & 0 & 0 & B_{m}^{T}PB_{m}-Q_{m} \end{array}\right]\bar{x}$$

where $\bar{x}$ = [*x*(*t*), *x*(*t *- *τ*_1_),..., *x*(*t *- *τ*_*m*_)]^*T *^and $\Xi =PA_{0}+A_{0}^{T}P+B_{0}^{T}PB_{0}+\sum_{k=1}^{m}Q_{k}$. If the LMI in (10) holds, then $E\frac{d}{dt}V(x(t))<0$, i.e., the linear stochastic gene network in (6) is asymptotically stable in probability.

### **Appendix C **Proof of Proposition 3

Consider the following equivalent equation

(C.1) $$E\int_{0}^{\infty }z_{o}^{T}(t)z_{o}(t)dt=EV(x(0))-EV(x(\infty ))+E\int_{0}^{\infty }\left(x^{T}(t)C^{T}Cx(t)dt+dV(x(t))\right)$$

where *V*(*x*(*t*)) > 0.

By the Itô formula, we get [49]

(C.2) $$\begin{array}{c} dV(x(t))={\left(\frac{\partial V(x(t))}{\partial x}\right)}^{T}(\sum_{k=0}^{m}f_{k}(x(t-\tau_{k}))dt+Gv(t)dt+\sum_{k=0}^{m}h_{k}(x(t-\tau_{k}))dW_{k}(t))\\ +\sum_{k=0}^{m}\frac{1}{2}h_{k}^{T}(x(t-\tau_{k}))\frac{\partial^{2}V(x(t))}{\partial x^{2}}h_{k}(x(t-\tau_{k}))dt \end{array}$$

Substituting the above equation into (C.1), by the fact that *V*(*x*(∞)) ≥ 0 and *W*_*k*_(*t*) is a zero mean Wiener process and independent of *x*(*t*), we get

(C.3) $$\begin{array}{c} E\int_{0}^{\infty }z_{o}^{T}(t)z_{o}(t)dt\leq EV(x(0))+E\int_{0}^{\infty }\left[x^{T}(t)C^{T}Cx(t\right)+{\left(\frac{\partial V(x(t))}{\partial x}\right)}^{T}(\sum_{k=0}^{m}f_{k}\left(x(t-\tau_{k})\right)+Gv(t))\\ +\sum_{k=0}^{m}\frac{1}{2}h_{k}^{T}(x(t-\tau_{k}))\frac{\partial^{2}V(x(t))}{\partial x^{2}}h_{k}(x(t-\tau_{k}))]dt \end{array}$$

By Fact 1, we have

(C.4) $$\begin{array}{c} {\left(\frac{\partial V(x(t))}{\partial x}\right)}^{T}Gv(t)=\frac{1}{2}{\left(\frac{\partial V(x(t))}{\partial x}\right)}^{T}Gv(t)+\frac{1}{2}v^{T}(t)G^{T}\left(\frac{\partial V(x(t))}{\partial x}\right)\\ \leq \frac{1}{4\gamma^{2}}{\left(\frac{\partial V(x(t))}{\partial x}\right)}^{T}GG^{T}\left(\frac{\partial V(x(t))}{\partial x}\right)+\gamma^{2}v^{T}(t)v(t) \end{array}$$

Therefore, we can obtain

(C.5) $$\begin{array}{ccc} E\int_{0}^{\infty }z_{o}^{T}(t)z_{o}(t)dt & \leq & EV(x(0))+E\int_{0}^{\infty }\left[x^{T}(t)C^{T}Cx(t\right)+{\left(\frac{\partial V(x(t))}{\partial x}\right)}^{T}(\sum_{k=0}^{m}f_{k}(x(t-\tau_{k})))\\ & + & \sum_{k=0}^{m}\frac{1}{2}h_{k}{\left(x(t-\tau_{k})\right)}^{T}\frac{\partial^{2}V(x(t))}{\partial x^{2}}h_{k}(x(t-\tau_{k}))\\ & + & \frac{1}{4\gamma^{2}}{\left(\frac{\partial V(x(t))}{\partial x}\right)}^{T}GG^{T}\left(\frac{\partial V(x(t))}{\partial x}\right)+\gamma^{2}v^{t}(t)v(t)]dt \end{array}$$

By the inequality in (16), we get

(C.6) $$E\int_{0}^{\infty }z_{o}^{T}(t)z_{o}(t)dt\leq EV(x(0))+\gamma^{2}E\int_{0}^{\infty }v^{T}(t)v(t)dt$$

Obviously, the filtering ability in (15) is achieved. Because the noise filtering ability is defined as the minimum effect of *v*(*t*) on the gene, so the noise filtering ability could be achieved by minimizing *γ *under the constraint of (16).

### **Appendix D **Proof of Proposition 5

Let us choose a Lyapunov function for gene network (25) as

$$V(x(t))=x^{T}(x)Px(t)+\sum_{k=1}^{m}\int_{t-\tau_{k}}^{t}x^{T}(s)Q_{k}x(s)ds>0$$

where *P *= *P*^*T *^> 0 and $Q_{k}=Q_{k}^{T}>0$. By Itô formula [49] and gene network (25), we get

$$\begin{array}{l} E\frac{dv(x(t))}{dt}=E\{{\left(\frac{\partial V(x(t))}{\partial x}\right)}^{T}[\sum_{i=1}^{L}\mu_{i}(z)\sum_{k=0}^{m}{\bar{A}}_{k,i}x(t-\tau_{k})\\ +\sum_{k=0}^{m}\Delta f_{k}(x)+\sum_{k=0}^{m}K_{k}\Delta g_{k}(x)]\\ +\sum_{k=0}^{m}(\frac{1}{2}{\left(\sum_{i=1}^{L}\mu_{i}(z)B_{k,i}x(t-\tau_{k})+\Delta h_{k}(x)\right)}^{T}\\ \times \frac{\partial^{2}V(x(t))}{\partial x^{2}}\left(\sum_{j=1}^{L}\mu_{j}(z)B_{k,j}x(t-\tau_{k})+\Delta h_{k}(x)\right))\} \end{array}$$

(D.1) $$\begin{array}{ll} = & E\{\sum_{i=1}^{L}\mu_{i}(z)\sum_{j=1}^{L}\mu_{j}(z)\{x^{T}(t)[P{\bar{A}}_{0,i}+{\bar{A}}_{0,i}^{T}P]x(t)\\ & +\sum_{k=1}^{m}\left[x^{T}(t)P{\bar{A}}_{k,i}x(t-\tau_{k}\right)+x^{T}(t-\tau_{k}){\bar{A}}_{k,i}^{T}Px(t)]\\ & +\sum_{k=1}^{m}\left[x^{T}(t)Q_{k}x(t)-x^{T}(t-\tau_{k})Q_{k}x(t-\tau_{k})\right]\\ & +\sum_{k=0}^{m}\left[x^{T}(t)P\Delta f_{k}(x)+\Delta f_{k}^{T}(x)Px(t)\right]\\ & +\sum_{k=0}^{m}\left[x^{T}(t)PK_{k}\Delta g_{k}(x)+\Delta g_{k}^{T}(x)K_{k}^{T}Px(t)\right]\\ & +\sum_{k=0}^{m}\left[x^{T}(t-\tau_{k})B_{k,i}^{T}PB_{k,j}x(t-\tau_{k}\right)\\ & +\Delta h_{k}^{T}(x)P\Delta h_{k}(x)+x^{T}(t-\tau_{k})B_{k,i}^{T}P\Delta h_{k}(x)\\ & +\Delta h_{k}^{T}(x)PB_{k,i}x(t-\tau_{k})]\}\} \end{array}$$

By Fact 1, we have

(D.2) $$x^{T}(t)P\Delta f_{x}(x)+\Delta f_{x}^{T}(x)Px(t)\leq \frac{1}{\alpha_{k,1}}x^{T}(t)PPx(t)+\alpha_{k,1}\Delta f_{k}^{T}(x)\Delta f_{k}(x),$$

(D.3) $$x^{T}(t)PK_{k}\Delta g_{k}(x)+\Delta g_{k}^{T}(x)K_{k}^{T}Px(t)\leq \frac{1}{\alpha_{k,3}}x^{T}(t)PK_{k}K_{x}^{T}Px(t)+\alpha_{k,3}\Delta g_{k}^{T}(x)\Delta g_{k}(x),$$

(D.4) $$\begin{array}{c} x^{T}(t-\tau_{k})B_{k,i}^{T}P\Delta h_{k}(x)+\Delta h_{k}^{T}(x)PB_{k,i}x(t-\tau_{k})\leq \frac{1}{\alpha_{k,2}}x^{T}(t-\tau_{k})B_{k,i}^{T}PPB_{k,i}x(t-\tau_{k})\\ +\alpha_{k,2}\Delta h_{k}^{T}(x)\Delta h_{k}(x), \end{array}$$

and

(D.5) 12xT(t−τk)Bk,iTPBk,jx(t−τk)+12xT(t−τk)Bk,jTPBk,ix(t−τk)≤12xT(t−τk)Bk,iTPBk,ix(t−τk)+12xT(t−τk)Bk,jTPBk,jx(t−τk)

for *k *= 0, 1,...,*m*, where *α*_*k*, 1_, *α*_*k*, 2_, and *α*_*k*, 3_, are positive constants.

Suppose the following inequality holds

(D.6) *P *<*βI*

where *β *is a positive constant. By (D.2)–(D.6) and (26), we get

(D.7) EdV(x(t))dt≤E{∑i=1Lμi(z){xT(t)[PA¯0,iT+A¯0,iTP+∑k=1mQk]x(t)+∑k=1m[xT(t)PA¯k,ix(t−τk)+xT(t−τk)A¯k,iTP(x(t)]+∑k=0m[xT(t−τk)Bk,iTPBk,ix(t−τk)]−∑k=1m[xT(t−τk)Qkx(t−τk)]+∑k=0m[αk,1ΔfkT(x)Δfk(x)+(αk,2+β)ΔhkT(x)Δhk(x)+αk,3ΔgkT(x)Δgk(x)]+∑k=0m[xT(t)[1αk,1PP+1αk,3PKkKkTP]x(t)]+∑k=0m[1αk,2xT(t−τk)Bk,iTPPBk,ix(t−τk)]}}<E{∑i=1Lμi(z)x¯T(Ξ11,i+Ξ12,iΞ22,i−1Ξ21,i+Ξ13,iΞ33,i−1Ξ31,i+Ξ14,iΞ44,i−1Ξ41,i)x¯}

where $\bar{x}$ = [*x*(*t*), *x*(*t*-*τ*_1_),⋯,*x*(*t*-*τ*_*m*_)]^*T *^and other matrices are defined in (27). If the following inequalities held

$$\Xi_{11,i}+\Xi_{12,i}\Xi_{22,i}^{-1}\Xi_{21,i}+\Xi_{13,i}\Xi_{33}^{-1}\Xi_{31,i}+\Xi_{14,i}\Xi_{44,i}^{-1}\Xi_{41,i}<0$$

for *i *= 1,...,*L*, then $E\frac{dV(x(t))}{dt}<0$. So the gene network (25) is asymptotically stable in probability.

### **Appendix E **Proof of Proposition 6

Consider the following equivalent equation

(E.1) $$E\int_{0}^{\infty }z_{o}^{T}(t)z_{o}(t)dt=EV(x(0))-EV(x(\infty ))+E\int_{0}^{\infty }\left(x^{T}(t)C^{T}Cx(t)dt+dV(x)\right)$$

where $V(x(t))=x^{T}(t)Px(t)+\sum_{k=1}^{m}\int_{t-\tau_{k}}^{t}x^{T}(s)Q_{k}x(s)ds$ for some *P *= *P*^*T *^> 0 and $Q_{k}=Q_{k}^{T}>0$.

By the Itô differential equation [49] and (29), we get

(E.2) $$\begin{array}{lll} dV(x(t)) & = & \{{\left(\frac{\partial V(x(t))}{\partial x}\right)}^{T}[\sum_{i=1}^{L}\mu_{i}(z)\sum_{k=0}^{m}{\bar{A}}_{k,i}x(t-\tau_{k})\\ & & +Gv(t)+\sum_{k=0}^{m}\Delta f_{k}(x)+\sum_{k=0}^{m}K_{k}\Delta g_{k}(x)]\\ & & +\sum_{k=0}^{m}(\frac{1}{2}{\left(\sum_{i=1}^{L}\mu_{i}(z)B_{k,i}x(t-\tau_{k})+\Delta h_{k}(x)\right)}^{T}\\ & & \times \frac{\partial^{2}V(x(t))}{\partial x^{2}}\left(\sum_{j=1}^{L}\mu_{j}(z)B_{k,j}x(t-\tau_{k})+\Delta h_{k}(x)\right))\} \end{array}$$

Substituting the above equation into (E.1), by the fact that $V(x(t))=x^{T}(t)Px(t)+\sum_{k=1}^{m}\int_{t-\tau_{k}}^{t}x^{T}(s)Q_{k}x(s)ds$ and *W*_*k*_(*t*) is a zero mean Wiener process and independent of *x*(*t*), we get

(E.3) $$\begin{array}{c} E\int_{0}^{\infty }z_{o}^{T}(t)z_{o}(t)dt\leq EV(x(0))+E\int_{0}^{\infty }\left\{x^{T}(t)C^{T}Cx(t\right)\\ +\sum_{i=1}^{L}\mu_{i}(z)\sum_{j=1}^{L}\mu_{j}(z)\{x^{T}(t)[P{\bar{A}}_{1,i}+{\bar{A}}_{1,i}^{T}P]x(t)\\ +\sum_{k=1}^{m}\left[x^{T}(t)P{\bar{A}}_{k,i}x(t-\tau_{k}\right)+x^{T}(t-\tau_{k}){\bar{A}}_{1,i}^{T}Px(t)]\\ +\sum_{k=1}^{m}\left[x^{T}(t)Q_{k}x(t\right)-x^{T}(t-\tau_{k})Q_{k}x(t-\tau_{k})]\\ +\sum_{k=0}^{m}\left[x^{T}(t)P\Delta f_{k}(x)+\Delta f_{k}^{T}(x)Px(t)\right]\\ +\sum_{k=0}^{m}\left[x^{T}(t)PK_{k}\Delta g_{k}(x\right)+\Delta g_{k}^{T}(x)K_{k}^{T}Px(t)]\\ +\sum_{k=0}^{m}[{\left(B_{k,i}x(t-\tau_{k})+\Delta h_{k}(x)\right)}^{T}\\ \times P(B_{k,j}x(t-\tau_{k})+\Delta h_{k}(x))]\}\\ +x^{T}(t)PGv(t)+v^{T}(t)G^{T}Px(t)\}dt \end{array}$$

By (D.2)–(D.6), (26) and $x^{T}(t)PGv(t)+v^{T}(t)G^{T}Px(t)\leq \frac{1}{\gamma^{2}}x^{T}(t)PGG^{T}Px(t)+\gamma^{2}v^{T}(t)v(t)$ (by Fact 1), we get

(E.4) $$\begin{array}{c} E\int_{0}^{\infty }z_{o}^{T}(t)z_{o}(t)dt\leq EV(x(0))+E\int_{0}^{\infty }\{\sum_{i=1}^{L}\mu_{i}(z){\bar{x}}^{T}[\Xi_{11,i}+\Phi +\Xi_{12,i}\Xi_{22,i}^{-1}\Xi_{21,i}\\ +\Xi_{13,i}\Xi_{33,i}^{-1}\Xi_{31,i}+\Xi_{14,i}\Xi_{44,i}^{-1}\Xi_{41,i}+\Xi_{15,i}\Xi_{55,}^{-1}\Xi_{51,i}]\bar{x}+\gamma^{2}v^{T}(t)v(t)\}dt \end{array}$$

By the the following inequalities,

(E.5) $$\Xi_{11,i}+\Phi +\Xi_{12,i}\Xi_{22,i}^{-1}\Xi_{21,i}+\Xi_{13,i}\Xi_{33,i}^{-1}\Xi_{31,i}+\Xi_{14,i}\Xi_{44,i}^{-1}\Xi_{41,i}+\Xi_{15,i}\Xi_{55,i}^{-1}\Xi_{51,i}<0$$

we get

(E.6) $$E\int_{0}^{\infty }z_{o}^{T}(t)z_{o}(t)dt\leq EV(x(0))+\gamma^{2}E\int_{0}^{\infty }v^{T}(t)v(t)dt$$

Obviously, the noise filtering ability in (15) is achieved. Furthermore, by Shur complement [35], the inequalities in (E.5) are equivalent to the LMIs in (31).

### **Appendix F **The design parameters of example 1

The parameters of the fuzzy model for the genetic regulatory network in (36)–(39) are listed in the following. Because the genetic regulatory network in (36)–(39) have two different time delays *τ*_1 _= 1 and *τ*_2 _= *τ*_3 _= 2, we can separate the system to *f*_0_(*x*(*t*)), *f*_1_(*x*(*t *- *τ*_1_)), and *f*_2_(*x*(*t *- *τ*_2_)). Therefore, we can obtain the the parameters of fuzzy model in (24) as follows

$$A_{0,i}=\left[\begin{array}{cccc} -1.0000 & 0 & 0 & 0\\ 0 & -0.1000 & 0 & 0\\ 0 & 0 & -10.0000 & 0\\ 0 & 0 & 0 & -2.0000 \end{array}\right],i=1,...,16$$

$$A_{1,1}=\left[\begin{array}{cccc} 0 & 0 & 0 & 0\\ 0.0014 & 0.0003 & -0.0016 & 0.0092\\ 0 & 0 & 0 & 0\\ 0 & 0 & 0 & 0 \end{array}\right],A_{1,2}=\left[\begin{array}{cccc} 0 & 0 & 0 & 0\\ -0.0120 & -0.0017 & -0.0190 & 0.0040\\ 0 & 0 & 0 & 0\\ 0 & 0 & 0 & 0 \end{array}\right],$$

$$A_{1,3}=\left[\begin{array}{cccc} 0 & 0 & 0 & 0\\ 0.0046 & 0.0008 & -0.0012 & 0.0013\\ 0 & 0 & 0 & 0\\ 0 & 0 & 0 & 0 \end{array}\right],A_{1,4}=\left[\begin{array}{cccc} 0 & 0 & 0 & 0\\ -0.0038 & -0.0008 & -0.0006 & 0.0010\\ 0 & 0 & 0 & 0\\ 0 & 0 & 0 & 0 \end{array}\right],$$

$$A_{1,5}=\left[\begin{array}{cccc} 0 & 0 & 0 & 0\\ 0.0015 & 0.0002 & -0.0017 & 0.0098\\ 0 & 0 & 0 & 0\\ 0 & 0 & 0 & 0 \end{array}\right],A_{1,6}=\left[\begin{array}{cccc} 0 & 0 & 0 & 0\\ -0.0132 & -0.0019 & -0.0198 & 0.0046\\ 0 & 0 & 0 & 0\\ 0 & 0 & 0 & 0 \end{array}\right],$$

$$A_{1,7}=\left[\begin{array}{cccc} 0 & 0 & 0 & 0\\ 0.0051 & 0.0006 & -0.0015 & 0.0016\\ 0 & 0 & 0 & 0\\ 0 & 0 & 0 & 0 \end{array}\right],A_{1,8}=\left[\begin{array}{cccc} 0 & 0 & 0 & 0\\ -0.0041 & -0.0004 & -0.0004 & 0.0011\\ 0 & 0 & 0 & 0\\ 0 & 0 & 0 & 0 \end{array}\right],$$

$$A_{1,9}=\left[\begin{array}{cccc} 0 & 0 & 0 & 0\\ 0.0013 & 0.0001 & -0.0064 & 0.0161\\ 0 & 0 & 0 & 0\\ 0 & 0 & 0 & 0 \end{array}\right],A_{1,10}=\left[\begin{array}{cccc} 0 & 0 & 0 & 0\\ -0.0121 & -0.0036 & -0.0312 & 0.0114\\ 0 & 0 & 0 & 0\\ 0 & 0 & 0 & 0 \end{array}\right],$$

$$A_{1,11}=\left[\begin{array}{cccc} 0 & 0 & 0 & 0\\ 0.0045 & 0.0010 & -0.0056 & 0.0046\\ 0 & 0 & 0 & 0\\ 0 & 0 & 0 & 0 \end{array}\right],A_{1,12}=\left[\begin{array}{cccc} 0 & 0 & 0 & 0\\ -0.0035 & -0.0004 & -0.0108 & 0.0051\\ 0 & 0 & 0 & 0\\ 0 & 0 & 0 & 0 \end{array}\right],$$

$$A_{1,13}=\left[\begin{array}{cccc} 0 & 0 & 0 & 0\\ 0.0013 & 0.0005 & -0.0082 & 0.0181\\ 0 & 0 & 0 & 0\\ 0 & 0 & 0 & 0 \end{array}\right],A_{1,4}=\left[\begin{array}{cccc} 0 & 0 & 0 & 0\\ -0.0135 & -0.0029 & -0.0353 & 0.0139\\ 0 & 0 & 0 & 0\\ 0 & 0 & 0 & 0 \end{array}\right],$$

$$A_{1,15}=\left[\begin{array}{cccc} 0 & 0 & 0 & 0\\ 0.0048 & 0.0016 & -0.0070 & 0.0056\\ 0 & 0 & 0 & 0\\ 0 & 0 & 0 & 0 \end{array}\right],A_{1,16}=\left[\begin{array}{cccc} 0 & 0 & 0 & 0\\ -0.0035 & -0.0015 & -0.0142 & 0.0067\\ 0 & 0 & 0 & 0\\ 0 & 0 & 0 & 0 \end{array}\right],$$

$$A_{2,1}=\left[\begin{array}{cccc} 0 & 0 & 0 & 0\\ 0 & 0 & 0 & 0\\ 0.9144 & 0.2136 & 0.0779 & 0.1045\\ -0.5708 & -0.0804 & -1.1212 & -0.0651 \end{array}\right],A_{2,2}=\left[\begin{array}{cccc} 0 & 0 & 0 & 0\\ 0 & 0 & 0 & 0\\ 1.0150 & 0.2319 & 0.0838 & 0.1052\\ -0.6193 & -0.0815 & -1.1619 & -0.0747 \end{array}\right],$$

$$A_{2,3}=\left[\begin{array}{cccc} 0 & 0 & 0 & 0\\ 0 & 0 & 0 & 0\\ 0.9885 & 0.2271 & 0.0786 & 0.1105\\ 0.3903 & 0.0707 & 0.0321 & 0.0587 \end{array}\right],A_{2,4}=\left[\begin{array}{cccc} 0 & 0 & 0 & 0\\ 0 & 0 & 0 & 0\\ 1.1017 & 0.2471 & 0.0851 & 0.1116\\ 0.4208 & 0.0817 & -0.0160 & 0.0436 \end{array}\right],$$

$$A_{2,5}=\left[\begin{array}{cccc} 0 & 0 & 0 & 0\\ 0 & 0 & 0 & 0\\ 0.9613 & 0.2367 & 0.0656 & 0.0882\\ -0.6307 & -0.0907 & -1.1712 & -0.0652 \end{array}\right],A_{2,6}=\left[\begin{array}{cccc} 0 & 0 & 0 & 0\\ 0 & 0 & 0 & 0\\ 1.0651 & 0.2556 & 0.0709 & 0.0898\\ -0.6861 & -0.1005 & -1.2153 & -0.0855 \end{array}\right],$$

$$A_{2,7}=\left[\begin{array}{cccc} 0 & 0 & 0 & 0\\ 0 & 0 & 0 & 0\\ 1.0376 & 0.2507 & 0.0671 & 0.0936\\ 0.4282 & 0.0545 & -0.0278 & 0.0713 \end{array}\right],A_{2,8}=\left[\begin{array}{cccc} 0 & 0 & 0 & 0\\ 0 & 0 & 0 & 0\\ 1.1552 & 0.2716 & 0.0736 & 0.0961\\ 0.4664 & 0.0519 & -0.0876 & 0.0393 \end{array}\right],$$

$$A_{2,9}=\left[\begin{array}{cccc} 0 & 0 & 0 & 0\\ 0 & 0 & 0 & 0\\ 0.9444 & 1.5569 & 0.1009 & 0.1353\\ -0.5787 & -0.1708 & -1.8931 & -0.1407 \end{array}\right],A_{2,10}=\left[\begin{array}{cccc} 0 & 0 & 0 & 0\\ 0 & 0 & 0 & 0\\ 1.0455 & 1.7747 & 0.1120 & 0.1330\\ -0.6337 & -0.1991 & -2.1010 & -0.1120 \end{array}\right],$$

$$A_{2,11}=\left[\begin{array}{cccc} 0 & 0 & 0 & 0\\ 0 & 0 & 0 & 0\\ 1.0188 & 1.7163 & 0.0989 & 0.1460\\ 0.3780 & 0.0803 & -0.7044 & 0.0626 \end{array}\right],A_{2,12}=\left[\begin{array}{cccc} 0 & 0 & 0 & 0\\ 0 & 0 & 0 & 0\\ 1.1328 & 1.9689 & 0.1082 & 0.1425\\ 0.3981 & 0.0719 & -0.8900 & 0.1078 \end{array}\right],$$

$$A_{2,13}=\left[\begin{array}{cccc} 0 & 0 & 0 & 0\\ 0 & 0 & 0 & 0\\ 0.7240 & 1.2753 & -0.0664 & -0.0897\\ -0.6473 & -0.1399 & -2.1535 & -0.1723 \end{array}\right],A_{2,14}=\left[\begin{array}{cccc} 0 & 0 & 0 & 0\\ 0 & 0 & 0 & 0\\ 0.8289 & 1.4911 & -0.0717 & -0.0945\\ -0.7167 & -0.1423 & -2.4263 & -0.1113 \end{array}\right],$$

$$A_{2,15}=\left[\begin{array}{cccc} 0 & 0 & 0 & 0\\ 0 & 0 & 0 & 0\\ 0.8011 & 1.4334 & -0.0708 & -0.0954\\ 0.4021 & 0.1288 & -0.9350 & 0.0494 \end{array}\right],A_{2,16}=\left[\begin{array}{cccc} 0 & 0 & 0 & 0\\ 0 & 0 & 0 & 0\\ 0.9204 & 1.6829 & -0.0797 & -0.1029\\ 0.4183 & 0.1612 & -1.1606 & 0.1453 \end{array}\right],$$

$$B_{0}={\left[\begin{array}{cccc} 0 & -0.1 & 0 & 0 \end{array}\right]}^{T},B_{1}={\left[\begin{array}{cccc} 0 & 0 & 0 & 0 \end{array}\right]}^{T},$$

$$B_{2}={\left[\begin{array}{cccc} 0 & 0 & 0 & 0.7 \end{array}\right]}^{T},G={\left[\begin{array}{cccc} 0 & 0.25 & 0 & 0.25 \end{array}\right]}^{T},$$

Because the function *f*(*x*(*t*)) is linear, the matrices *A*_0,*i *_are the same.

## Authors' contributions

BSC gives the topic and derives some results, and YTC gives some proofs of the results and performs the example simulations.
